# Diffusion-Based Frequency Hopping for Collision Mitigation in Dense Bluetooth Networks

**DOI:** 10.3390/s25185893

**Published:** 2025-09-20

**Authors:** Giwon Yang, Hyungjoon Shin, Hyogon Kim

**Affiliations:** Department of Computer Science and Engineering, Korea University, Anam-Dong, Sungbuk-Gu, Seoul 02841, Republic of Korea; giwone@korea.ac.kr (G.Y.); kilk119@korea.ac.kr (H.S.)

**Keywords:** Bluetooth, frequency hopping, random access MAC, diffusion theory, packet collision, mean first encounter time (MFET)

## Abstract

This paper challenges the conventional wisdom of using uniform random resource selection for collision resolution in distributed scheduling, particularly in wireless protocols. Bluetooth, being one such technology, is analyzed through its frequency hopping mechanism to explore for a better alternative in random access MAC (medium access control). Using diffusion theory, we characterize Bluetooth’s original frequency hopping as exhibiting maximum diffusivity, which correlates with unnecessarily high collision rates and a short mean first encounter time (MFET) between nodes. MFET, defined as the expected time until two independent hopping sequences first collide on the same channel, serves as an intuitive metric for evaluating collision likelihood. This insight leads to the proposal of a new collision avoidance mechanism with reduced diffusivity, effectively increasing MFET while maintaining efficient spectrum utilization. Our analysis and simulation results demonstrate that it can significantly lower packet collisions, outperforming existing techniques such as adaptive frequency hopping. The results are further corroborated by a real-life prototype implementation that closely replicates the predicted performance. The proposed diffusion-based MAC, by explicitly targeting longer MFETs, is expected to better handle dense Bluetooth environments, which are becoming increasingly common.

## 1. Introduction

Many distributed medium access control (MAC) protocols for shared-medium technologies, particularly wireless protocols, employ random access as a means of resolving collisions in resource scheduling. When a random access scheme is employed, the most common choice is the uniform random distribution, as seen in Bluetooth frequency hopping [1], IEEE 802.11 CSMA/CA [2], or the distributed sidelink resource scheduling in recent 3GPP releases [3], among many others. In this paper, we question the accepted practice of using the uniform random distribution for resolving resource collisions. Specifically, we draw upon the theory of diffusion [4] to provide a perspective on how desirable the uniform randomness is to mitigate resource collisions. Our objective is to investigate if there is a better random scheduling method by using Bluetooth frequency hopping as a case study.

More than two decades after its introduction, Bluetooth devices continue to proliferate. Not only are wireless headsets and earbuds becoming the norm, but industrial applications are also expanding. For instance, tire pressure monitoring systems (TPMSs) and other sensors on trucks are increasingly being connected through Bluetooth technology. As Bluetooth devices become more pervasive in everyday life, the likelihood of encountering a large number of them in close proximity is increasing. For example, multiple users may be wearing Bluetooth earbuds or headsets within the typical Bluetooth communication range (e.g., 10 m) in a crowded subway car. In fact, the interference issue was anticipated and investigated in the early days of Bluetooth development [5,6,7,8,9]. However, given the widespread real-world adoption of Bluetooth, now is an opportune time to revisit interference mitigation strategies for densified environments. In this paper, we demonstrate that there is indeed room for further improvement.

Originally, Bluetooth Classic employed frequency hopping that uniformly selected among 79 channels at 1600 Hz to minimize the impact of interference from other technologies such as Wi-Fi that use the same unlicensed band in the 2.4 GHz range. A more advanced mechanism, adaptive frequency hopping (AFH), was later introduced in Bluetooth v1.2 [10]. AFH enables Bluetooth devices to monitor, identify, and dynamically exclude channels subject to static interference. AFH can reduce the number of used (“good”) channels from 79 to as few as 20 in the worst case, after which uniform random hopping is performed within the reduced channel set. However, this reduction in available channels can exacerbate the collision problem among Bluetooth piconets competing for limited spectrum resources.

In subsequent discussions, we reveal that current Bluetooth frequency hopping operates at the high end of the diffusive spectrum, exhibiting maximum diffusivity. However, diffusion theory predicts that greater diffusivity leads to more frequent collisions. This insight introduces a new design space in which diffusivity in frequency hopping can be controlled to improve collision mitigation in Bluetooth. Unlike the conventional uniform random method, our proposed approach retains and learns from past collision experiences through reinforcement learning. This “learned memory” is implemented in the form of a Q-table, which increases the value of channels that experience fewer collisions. Crucially, our DFH-RL further limits the hopping to a learned "neighborhood" of channels, which constrains the hopping range ($H_{max}$) rather than spanning the entire channel space. This adaptive learning process naturally increases the distance between piconets, which achieves an increase in MFET and collision reduction effects suggested by diffusion theory.

We propose a novel mechanism that implements diffusive frequency hopping and demonstrate through analysis, simulation, and prototype implementation that it significantly reduces collisions compared to the existing Bluetooth standard. When combined with multi-agent reinforcement learning (MARL), the system autonomously distributes piconets across distinct frequency subsets, within which they perform diffusive hopping. Even in scenarios with a large number of contending piconets and a limited number of available channels, the diffusive algorithm substantially reduces collisions, achieving up to a 62% reduction in packet collision probability (approximately 2.7-fold) compared to the Bluetooth standard.

To enhance Bluetooth performance in increasingly high-density environments, optimizing the frequency hopping algorithm will continue to be an important area of research and development. Continual optimization is actively pursued by industry leaders in various sectors. For instance, Apple (Cupertino, CA, USA) designed a custom W1/H1 chip with optimized frequency hopping and interference mitigation to ensure smooth Bluetooth audio even in noisy environments (e.g., subways and conferences). Siemens implemented proprietary frequency hopping algorithms in its Industrial Wireless LAN (IWLAN) solutions for factory automation. These algorithms reduce packet loss and latency in metal-rich, multipath-prone environments, where off-the-shelf Wi-Fi fails. Zebra optimized Bluetooth hopping to avoid interference from Wi-Fi in retail stores. It helped maintain low-latency scanning, which is essential for real-time inventory and checkout. These successful and continuing innovations motivate us to investigate the possibility of even more effective interference mitigation techniques in this paper.

The rest of this paper is organized as follows. Section 2 briefly surveys prior work on self-interference among Bluetooth piconets, both before and after the introduction of AFH. Section 3 examines the problem of the high packet collision probability in the original uniform random hopping in Bluetooth. Section 4 models frequency hopping as a diffusion process and explains why Bluetooth frequency hopping suffers from high self-interference. It identifies more desirable diffusion parameters for collision mitigation, implements a diffusive hopping algorithm, and evaluates its performance in comparison to the current standard. Section 6 addresses related concerns such as friendliness to legacy hopping methods, coexistence with Wi-Fi, and robustness to jamming. Finally, Section 7 concludes this paper.

## 2. Related Work

While extensive research has focused on mitigating Wi-Fi interference in Bluetooth devices, significantly less work has addressed self-interference between Bluetooth piconets.

The issue of self-interference among Bluetooth piconets was first identified as early as 2000 [5]. El-Hoiydi [6] analyzed this problem, deriving an upper bound on the packet error rate and a lower bound on Bluetooth Classic link throughput under interference from other piconets. Arumugam et al. [7] examined the impact of interference between Bluetooth-enabled consumer electronic devices in home and office environments. In response, Jiang et al. [8] proposed a hop set partitioning scheme to reduce collisions. By partitioning the original Bluetooth hop band into five orthogonal hop sets and having each central device randomly and independently select one, they demonstrated a system throughput improvement of over 10% over the original Bluetooth single-hop-set scheme. Similar proposals of artificially dispersing piconets across hop sets followed. However, restricting piconets from certain frequency ranges does not maximize channel resource utilization.

After AFH was standardized, Popovski et al. [9] proposed an adaptive self-allocation scheme, where devices select a subset of frequency channels to minimize interference. However, it is argued that due to the limited bandwidth and the restriction on the minimum hop set size, these methods degrade rapidly in multi-piconet environments [11]. Another category of strategies improves AFH by optimizing channel utilization [12]. Instead of entirely excluding “bad” channels from the hop set, these strategies would assign different usage probabilities to channels based on observed conditions. In a broader context, sensing interference that has certain patterns, such as those generated by radar, better by identifying such interference has recently been proposed [13] by excluding the predicted interfered channels from the used channels to improve the communication performance. This helps to evade the jamming signal [14]. Using neural networks to recognize patterned interference [15] also falls into this category. Finally, Li et al. [16] proposed transmitting the same packet simultaneously on two distinct frequency-hopped channels. Their results showed that this technique significantly lowers the packet error rate when only a few piconets coexist. However, beyond the hardware modification costs, this approach could cause channel congestion in dense deployments due to the doubled bandwidth usage.

A few non-uniform random hopping mechanisms for Bluetooth have recently been investigated. Ganipsetty et al. [17] used a regular, periodic pattern to obtain a higher signal-to-interference ratio (SNR) and lower bit error ratio (BER). Eltholt [18] proposed a chaotic frequency hopping method for better co-existence with Wi-Fi. But this work produced visible improvement under a very large number of piconets, up to 40, and with a very small number of channels, such as ten.

In addition to the above, recent studies have proposed new approaches to mitigate the limitations of AFH in dense Bluetooth environments. For instance, eAFH [19] introduces an informed exploration mechanism that accelerates the reintegration of previously excluded channels, thereby maintaining channel diversity under dynamic interference. However, it still inherits AFH’s reliance on pseudo-uniform random hopping, which constrains its ability to mitigate self-interference. Chaotic hopping [20] has also been explored to enhance resilience against jamming attacks by generating highly unpredictable hopping sequences, but its primary objective is focused on security against external adversaries rather than minimizing collisions among friendly piconets.

Beyond Bluetooth-specific mechanisms, a variety of intelligent MAC protocols have been investigated in the broader IoT and ad hoc networking domains. For example, Z-MAC [21] combines CSMA and TDMA to balance contention-based flexibility with scheduled efficiency, showing improved throughput in dense wireless sensor networks. However, it requires global slot synchronization, rendering direct application to Bluetooth infeasible. Similarly, Dynamic Nexus Mesh Communication (DNMC) [22] leverages edge computing to support decentralized routing and resource allocation in dense urban IoT deployments, but its design assumes mesh topologies and additional infrastructure support. Reinforcement learning-based MAC protocols, such as DQN-optimized MAC [23], adapt channel access policies in real time to reduce collisions, yet they demand substantial computational resources, which are not available in lightweight Bluetooth controllers. Finally, multi-objective optimization frameworks [24] ensure fairness and minimum channel capacity allocation among heterogeneous IoT devices, but they typically require centralized coordination and extensive global knowledge. Nevertheless, when applied to Bluetooth, these approaches still operate within AFH’s pseudo-uniform random hopping structure, which limits their effectiveness in mitigating self-interference. Furthermore, many rely on centralized decision-making, making them unsuitable for Bluetooth’s inherently distributed nature that depends solely on local information.

The dearth of research on Bluetooth self-interference and the recent gap in the literature suggest an over-reliance on AFH’s perceived effectiveness. However, as we will show, AFH is primarily designed to mitigate static interference and offers limited protection against self-interference. In contrast to previous studies on self-interference, this paper introduces a theoretical framework for characterizing frequency hopping, along with a practical design that leverages reduced diffusivity to achieve significant performance gains, which is proven by a proof-of-concept (POC) prototype. We show that the diffusion theory-based non-uniform hopping scheme combined with reinforcement learning remains effective across a wide range of competing Bluetooth network populations. Table 1 summarizes the more recent related work since 2020.

Despite this extensive body of work, three critical gaps remain:

- Lack of predictive analysis: Existing methods cannot predict collision probability analytically before collisions occur; they rely on empirical measurement or reactive adaptation.
- Absence of systematic optimization: Current approaches adjust hopping behavior heuristically or through trial-and-error learning, without a unifying theoretical model.
- Insufficient scalability insights: Prior work does not provide analytical explanations of how collision probability scales with increasing device density, a crucial requirement for next-generation dense Bluetooth environments.

To highlight the distinctions, Table 2 contrasts heuristic methods, standalone RL approaches, and our proposed diffusion-based frequency hopping with RL. Our work addresses these gaps by introducing diffusion theory as a theoretical foundation and combining it with reinforcement learning for real-time adaptability. Diffusion theory provides the analytical framework to predict collision probabilities, optimize hopping dynamics via diffusion coefficient control, and derive scalability insights for ultra-dense scenarios. Reinforcement learning complements this by adapting hop decisions in real time under dynamic conditions. Together, this synergy uniquely equips our approach to deliver both predictive analytical rigor and practical adaptability, bridging the limitations of purely heuristic or purely RL-based solutions.

## 3. Problem Description

**Figure 1:**
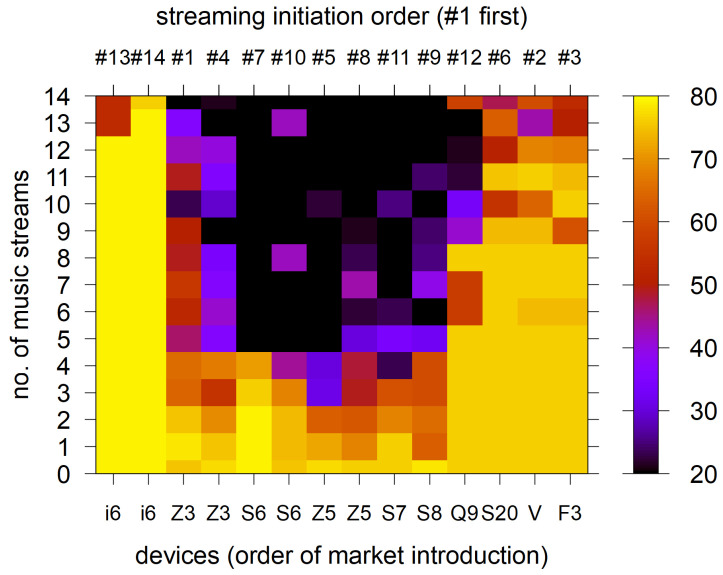
Number of “good” channels maintained by the piconets as music streams increase. Bottom x-axis shows devices by market introduction (left to right). Top x-axis shows the streaming initiation order (#1 = first). Device names are abbreviated (e.g., i6 and Z3); full model names are given in Table 3.

**Figure 2:**
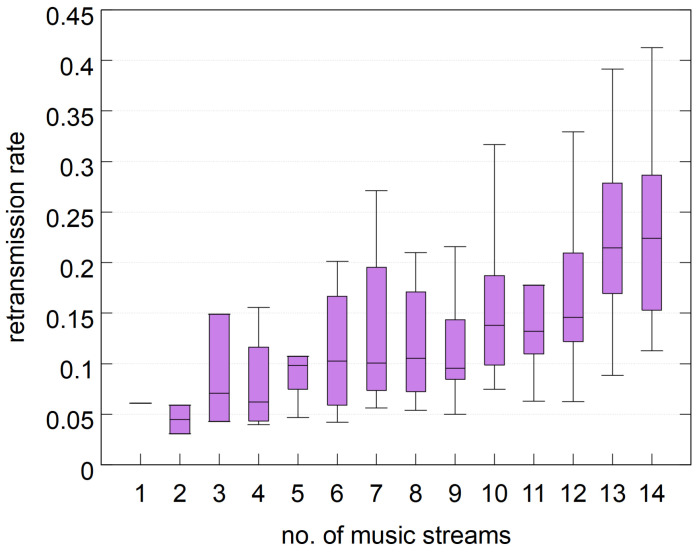
Average packet retransmission event rate.

**Figure 3:**
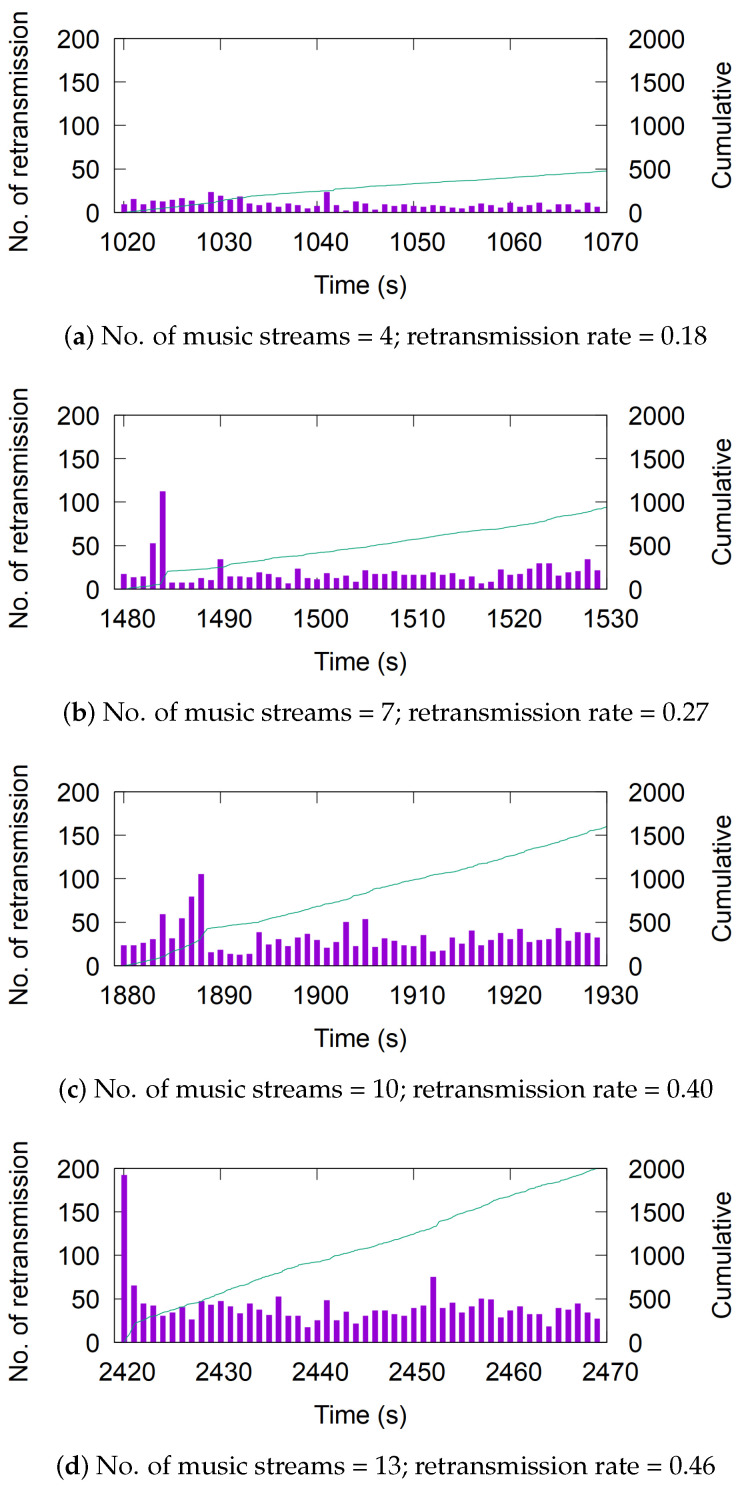
The number of retransmissions per second experienced by F3 as music streams increase with time. Purple bars indicate per-second retransmission counts, and the green solid line shows cumulative retransmissions.

To motivate this investigation, we measured the extent of channel space reduction under AFH due to self-interference. Figure 1, Figure 2 and Figure 3 present the measurement results obtained using an Ellisys Bluetooth protocol analyzer [25]. The measurements were conducted in a radio frequency (RF)-shielded room to focus only on the self-interference, blocking other radio technologies such as Wi-Fi. We marshaled 14 smart phones released over the last decade, each implementing its own vendor-specific AFH algorithm, as AFH implementation is not standardized. Then we formed 14 independent piconets, each consisting of a smartphone as the central device and a headset as the peripheral. Immediately after the piconet formation, there is no active music stream. Next, we sequentially initiated music streaming on the 14 piconets, adding one stream per piconet at a time. Each time a music stream was added, this condition was maintained for 100 s, and the AFH channel map and packet retransmission changes were analyzed using the air capture data collected with the Ellisys Bluetooth protocol analyzer. The smartphone streams the music to the headset using SBC (Sub-Band Codec) or AAC (Advanced Audio Codec) as default, depending on the implementation of the smartphone. Figure 1 illustrates how the number of usable channels in the channel map (i.e., the set of available channels) decreases over time as additional music streams are introduced (from bottom to top). The devices are arranged in order of their market introduction over seven years (from left to right; see also Table 3).

The result reveals that the channel map reduction is not uniform across piconets but varies significantly, reflecting the diversity of AFH implementations. More recent models, such as the Z Flip 3 (“F3”), tend to retain almost all channels in their maps, similar to the earliest model in our set, the iPhone6 (notably, iPhones are known not to exclude externally interfered channels [19]). In contrast, some devices reduce the number of available channels to the minimum, i.e., 20 channels.

Inadequate interference mitigation due to suboptimal AFH increases overall interference between piconets, leading to a higher probability of packet loss and retransmissions. This, in turn, can degrade the quality of experience (QoE). Figure 2 provides evidence of this effect by showing the number of packet retransmission events per packet transmission during the shielded room experiment (where a single event may involve multiple retransmissions per packet). The results clearly indicate an increasing retransmission ratio as the number of streams rises, reaching over 20% when the number of piconets is large.

For instance, the piconet created by F3 experienced the highest number of retransmissions, as shown in Figure 3. When competing with 10 or more active music streams, its retransmission rate exceeds 40%. Such a high packet loss rate can significantly impact user experience for various applications. Notably, this particular device has been introduced to the market recently, suggesting that newer devices may still be susceptible to suboptimal interference mitigation implementations.

The measurement results reveal that the effectiveness of interference mitigation through frequency hopping is surprisingly inadequate, potentially undermining advancements in other related technologies. For instance, Bluetooth audio codecs have evolved from SBC to AAC, AptX, and LDAC (Lossless Digital Audio Codec) to enhance user experience, requiring an increasingly larger bandwidth. However, without effective interference mitigation and efficient channel utilization, their full performance potential may not be realized, particularly in future dense deployment environments. Clearly, AFH implementations must first be improved. However, as we will later demonstrate, even an optimally implemented AFH has a fundamental limitation due to its reliance on uniform random channel hopping. A theoretical analysis reveals a design space where interference mitigation can be significantly improved. Moreover, this theory provides a foundation for designing an enhanced frequency hopping mechanism. In the remainder of this paper, we discuss the theory, implementation, and properties of this new frequency hopping mechanism.

## 4. Diffusion-Based Frequency Hopping

To gain theoretical insights into the fundamental limitations of uniform random frequency hopping in the current Bluetooth specification, we draw on diffusion theory. Unlike conventional heuristic or reactive methods, diffusion theory provides a superior theoretical foundation by offering the following:

- Predictive Analytical Capability for Collision Probability: Unlike simple probabilistic models that rely on static assumptions, diffusion theory enables a dynamic and predictive analysis of collision probability over time. By modeling frequency channels as a one-dimensional space and frequency hoppers (devices) as particles, diffusion equations can be used to predict the probability distribution of a device’s location (i.e., which channel it is in) over time. This allows for the calculation of the mean first encounter time (MFET) as a function of various parameters, such as hopping rate, number of devices, and initial channel distribution, rather than just a static value.
- Systematic Optimization Framework through Diffusion Coefficient Control: Diffusion theory provides a systematic optimization framework by controlling the "diffusion coefficient." This coefficient, which represents the rate of particle diffusion, can be mapped to controllable system parameters, such as the randomness of the hopping sequence or the aperiodicity of the hopping algorithm. By manipulating this coefficient, the system can actively steer the frequency hopping process to minimize the probability of "collisions," which occur when devices occupy the same channel simultaneously.
- Scalability Insights for Dense Network Deployments: For dense network deployments, diffusion theory offers powerful scalability insights. While conventional methods struggle to predict performance in high-density scenarios, diffusion theory can provide an analytical approximation for how the collision probability scales with the number of piconets. By treating the system as a continuous medium, it becomes possible to analyze system behavior as the density of “hoppers” increases, providing a theoretical performance upper bound that guides the design of scalable and robust protocols for future ultra-dense environments.

Building on this foundation, we design and implement a diffusion-based channel hopping scheme in this section. To the best of our knowledge, diffusion theory has not previously been applied to model or improve random access in Bluetooth or in other wireless MAC protocols.

### 4.1. Frequency Hopping as Diffusion: Model

The frequency hopping of independent piconets and the resulting packet collisions in a channel can be effectively modeled as the encounter of diffusing particles. Independent piconets correspond to independent diffusing particles, channel space to a one-dimensional diffusion interval *L*, channel hopping distance to the diffusion length Δx, slot time to the diffusion time unit Δt, and packet collisions to particle encounters. Furthermore, Bluetooth frequency hopping can be represented as diffusion with periodic boundaries, as the channel index wraps around modulo *L* (modL). Table 4 summarizes the analogy between diffusion and Bluetooth frequency hopping.

For the diffusion of two particles in a finite domain with periodic boundary conditions, the encounter problem simplifies to analyzing their relative coordinates, with an effective diffusivity equal to the sum of their individual diffusion constants. We can assume one particle is fixed while the other diffuses with diffusivity $D=2D_{1}$, where $D_{1}$ is the diffusion constant of a single particle in one dimension [26], i.e.,

(1) $$D_{1}=\frac{\langle {\left(\Delta x\right)}^{2}\rangle }{2\Delta t}.$$

The problem is thus equivalent to the first-passage problem for a single random walker. Then, Tejedor et al. demonstrated that the mean first encounter time (MFET) for a continuous-time, continuous-space random walk in an interval is

(2) $$E\left[t_{c}\right]=\frac{1}{2D}d\left(L-d\right),$$

where *d* denotes the initial displacement between the two particles [4]. We use Equation (2) to predict the average packet collision probability per time slot Δt in Bluetooth, considering different diffusion constants $D_{1}$. This diffusivity in channel hopping introduces a new dimension for exploring an improved frequency hopping method.

One caveat in equating the MFET $E[t_{c}]$ to the inverse of the packet collision probability, i.e., $P_{col}=1/E\left[t_{c}\right]$, is that in a conventional diffusion system, particles remain in close proximity after their first encounter. This is why Equation (2) represents the time to the first encounter. Unlike Bluetooth, diffusing particles will encounter each other more frequently after the initial encounter. To account for this difference, we modify the diffusion model to reset with a new random displacement $d^{\prime }$ after each encounter. This ensures that the assumptions of Equation (2) hold repeatedly. This behavior naturally applies to standard Bluetooth hopping, as Bluetooth piconets randomly select the next channel over the entire interval *L*. We also impose the same reset condition in our proposed alternative frequency hopping scheme after a collision.

**Figure 4:**
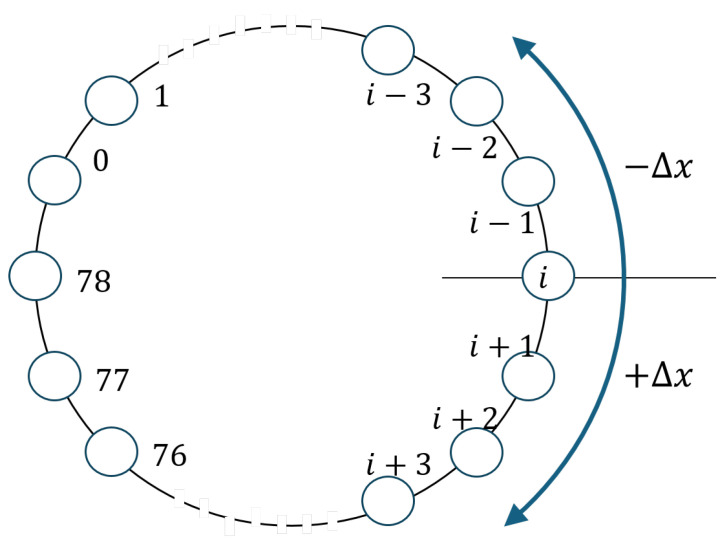
Hopping on Bluetooth channel space; L=79.

A direct implication of Equation (2) is that increasing the MFET (i.e., decreasing $P_{col}$) requires reducing *D*. According to Equation (1), this can be achieved by decreasing Δx for a given Δt or increasing Δt. In the proposed diffusive frequency hopping scheme, we leverage both approaches. If we model the Bluetooth channel space as a ring topology where hopping occurs (Figure 4), standard frequency hopping exhibits maximum diffusivity, as a piconet can hop from the current channel *i* to any other channel. The hop distance can be as large as half the channel space, i.e., Δx≤⌊L/2⌋. Consequently, diffusion theory predicts the shortest $E[t_{c}]$, leading to the highest $P_{col}$ in standard frequency hopping. This motivates the exploration of an alternative—a more diffusion-like frequency hopping scheme that constrains Δx to a smaller value, thereby reducing $P_{col}$. This approach restricts hopping to a local neighborhood of channels until a collision occurs, at which point a channel reset is triggered.

Bluetooth frequency hopping is not a continuous-time, continuous-space random walk as in theoretical diffusion. Instead, we approximate it using discrete movements that average to Δx per slot time. Since frequency hopping occurs at each slot time, it can be modeled as a one-dimensional discrete-time, discrete-space random walk $\left(\ldots ,a_{k},a_{k+1},\ldots \right)$ with periodic boundary conditions [27], where $\bar{a_{i}}=\Delta x$. In the proposed diffusive hopping scheme, which discretely approximates diffusion, we introduce a parameter $H_{max}$ to define the maximum hop distance, where $\Delta x=\frac{H_{max}+1}{2}$. For example, when $H_{max}=2$, the instantaneous hopping distance is dx∈{−2,−1,1,2}, yielding an expected hop distance of 〈dx〉=Δx=1.5. A neighboring piconet exhibits similar movement but with a relative offset *d*, modeled as $\left(\ldots ,d+b_{k},d+b_{k+1},\ldots \right)$, where $\bar{b_{i}}=\Delta x$ as well. A collision occurs when these two piconets’ random walks eventually intersect, overcoming the initial displacement *d*. Unlike prior work on hop sets [8,9], the diffusive hopping scheme does not forcibly restrict piconets to particular subsets of channels.

**Figure 5:**
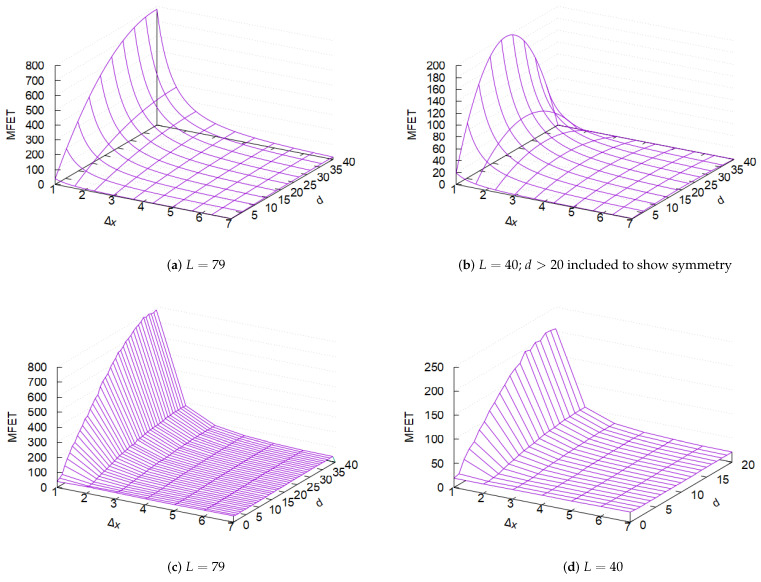
Mean first encounter time (MFET) between two piconets with diffusive frequency hopping as continuous-time, continuous-space diffusion (*L*: channel space size; Δx: channel hopping distance; *d*: initial channel separation); (**a**,**b**) analytical results and (**c**,**d**) simulation results for L=79 and L=40, respectively.

Figure 5a,b presents the MFET predicted by the theoretical model for different channel space sizes *L*, considering n=2 piconets under diffusion as a function of Δx and *d*. The MFET is measured in rounds, where each round *r* consists of unsynchronized frequency hops by the two piconets. The length of a round satisfies σ≤|r|≤2σ, where σ represents two slots—one for a central transmission and the other for a peripheral transmission. The figures highlight the critical role of small Δx, as MFET remains minimal regardless of *d* when Δx is large. However, when Δx is small, *d* has a significant impact. In particular, Figure 2b confirms Equation (2), which predicts that d=L/2 maximizes MFET for a given *D*. Unfortunately, *d* is not a parameter that a single piconet can explicitly control. The size of the ring *L* also plays a crucial role; for instance, when L=40, MFET decreases by a factor of 4 compared to L=79. The key takeaway from this theoretical analysis is that Δx should be kept small for diffusion-based frequency hopping to significantly extend the collision time $E[t_{c}]$, compared to the uniform random hopping used in standard Bluetooth.

Figure 5c,d presents the MFET observed in simulations. Despite deviations from the continuous-time, continuous-space model, the qualitative trends predicted by the theory are reproduced in the discrete simulation. However, some quantitative differences are noticeable—specifically, the minimum MFET is not as low as the analytical model predicts. For instance, even as diffusivity (i.e., increasing Δx) is maximized, the MFET for L=79 does not drop below 40 rounds. This discrepancy arises because, in the analytical model, Δx is treated as an infinitesimally small, fixed quantity, whereas in simulations, it represents the mean of integer-valued frequency movements dx. With the same Δx for two piconets, the analytical model predicts a collision, but in practice, the system can often avoid collisions.

Finally, the MFET equation can be generalized to multiple particles (e.g., piconets) as $E\left[t_{c}\right]\approx \frac{L^{2}}{2nD}$ when *n* is very large [28]. However, this broad approximation does not fully meet our needs for two key reasons. First, the number of piconets *n* is constrained by spatial limitations (e.g., the number of earbuds worn by passengers in a subway car) and does not grow arbitrarily large. Second, unlike Equation (2), this equation entirely ignores *d*. Thus, we turn to simulation to evaluate the proposed scheme in the next section. Equation (2) suggests that to maximize MFET in frequency hopping, *L* should be large, Δx should be small, and *d* should be maximized. While *L* and Δx can be set as system parameters, *d* must dynamically adapt as other piconets enter and leave the communication range. Specifically, piconets should be evenly distributed across the channel space. To achieve this, we integrate the diffusive hopping scheme with a reinforcement learning (RL)-based algorithm that automatically maintains channel separation from other diffusively hopping piconets in the proximity.

### 4.2. DFH Design and Implementation Considerations

Here, we design the diffusion-based frequency hopping (DFH) algorithm for Bluetooth. As discussed above, we integrate multi-agent reinforcement learning (MARL) into DFH, allowing each piconet to select the best channel to hop to, indirectly increasing *d*. For convenience, we refer to the proposed scheme as DFH-RL (diffusion-based frequency hopping with RL). For the RL component, we adopt simple single-state Q-learning [29], a model-free, off-policy, and temporal difference method that does not require a model of the environment, making it suitable for rapidly changing, partially observable, and distributed wireless systems. It has low computational and memory complexity, maintaining one Q-value per available action with constant-time updates, while still enabling adaptive learning through the discount factor. This choice is motivated by the absence of inherent value associated with individual channels. Since channel allocations change every two slots (e.g., 1.25 ms), they are too transient to be effectively incorporated into a full Q-learning framework. Additionally, in our distributed system, obtaining global information is challenging. Another advantage of single-state Q-learning is its lower complexity, which enhances adaptability. Unlike SARSA [30], which is on-policy and may adapt more conservatively, Q-learning’s off-policy updates accelerate convergence. Compared to deep RL methods, it avoids high computational and memory overhead, enabling lightweight firmware-level implementation. In this paper, we utilize the single-state Q-learning framework to leverage historical system information [29]. The action space corresponds to the set of available channels. For rewards, we use a simple assignment: at each round, R=0 for a successful transmission and R=−1 for a collision. Since the number of piconets involved in a collision cannot be distinguished, every collision is penalized equally with a unit negative reward.

Algorithm 1 presents the implementation of DFH-RL. It follows an ϵ-greedy exploration strategy, where ϵ represents the explore/exploit threshold. Other parameters are defined as follows: *Q* is the Q-table, $N_{a}$ is the number of agents (piconets), $CH_{cur}$ is the current channel, and *A* is the set of agents. The learning rate is denoted by α, and γ is the discount factor for the reward. The Q-table values are initialized to zero, ensuring that unexplored channels receive higher initial values than channels where the piconet has experienced collisions.
**Algorithm 1** Hopping policy Q-learning.  1:**procedure** cal_reward(A,a,x)                ▹ Reward calculation  2:      **for** $1\leq i\leq N_{a}$ **do**                      ▹$N_{a}$: no. of agents  3:            **if** ($i\neq a_{id}$ and $A\left[i\right].CH_{cur}=x$) **then**  4:                     ▹*x*: action, $CH_{cur}$: channel being used  5:                col←col||1                   ▹col: collision flag  6:            **end if**  7:      **end for**  8:      return −col  9:**end procedure**10:** **11:**procedure** update_q_table(*a*, *x*, *R*)              ▹ Q-table update12:      best_nxt←select_action(a)          ▹ LFH-RL, AFH-RL or DFH-RL13:      a.Q[x]←a.Q[x]+α(R+γ·a.Q[best_nxt]−a.Q[x])14:**end procedure**15:** **16:**procedure** run_RL17:      **for** 1≤round≤MAX **do**18:            ▹ Each piconet selects next channel once in each round19:           **for** $1\leq i\leq N_{a}$ **do**20:               action←select_action()        ▹ LFH-RL, AFH-RL or DFH-RL21:               R←cal_reward(A,A[i],action)22:               update_Q(A[i],action,R)23:               $A\left[i\right].CH_{cur}\leftarrow action$24:           **end for**25:     **end for**26:**end procedure**

To implement this algorithm on Bluetooth devices, we must address two challenges arising from the reinforcement learning component in DFH-RL.

#### 4.2.1. Exploitation—Q-Table Synchronization

When DFH-RL is not performing ϵ-greedy exploration, it selects a channel based on Q-table values. If the central and peripheral update the Q-table independently based on their respective measurements, discrepancies may arise due to their differing perspectives on collision detection. For example, when the central transmits data, the peripheral may be engaged in multi-pairing with another central. In this scenario, the central might interpret the lack of a response as a collision, while the peripheral—unaware of the transmission—does not perceive a collision. Consequently, their evaluations of the selected channel may become unsynchronized. If this occurs, they may exploit different channels in the next round, breaking the connection. To prevent this issue, the Q-table must be explicitly synchronized between the central and peripheral.

Instead of transmitting the entire Q-table, it is sufficient for the central to send only the best channel number to the peripheral. In this paper, the central communicates the best channel for the peripheral to exploit for the next τ seconds, which the peripheral must acknowledge. In this paper, we set τ=2. Additionally, a mechanism is needed to handle cases where the Q-table cannot be updated. For instance, static interference or jamming may prevent the update message from being transmitted, requiring a fallback strategy. To address this, if either the central or peripheral fails to respond or acknowledge within a specific timeframe, the system temporarily switches from DFH-RL to AFH. During this period, new Q-value updates are performed before reverting to DFH-RL.

#### 4.2.2. Exploration—Pseudo-Random Number Sequence Synchronization

In DFH-RL, ϵ-greedy exploration selects channels uniformly at random but with reduced diffusivity. To ensure both the central and peripheral choose the same next channel during diffusive exploration, they must use the same random number. To achieve this, DFH-RL utilizes the standard Bluetooth hopping kernel twice: once for the exploration/exploitation decision and once for determining the diffusive displacement (dx). The former uses the central’s Bluetooth device address and central’s clock as parameters, while the latter relies on the Peripheral’s device address.

**Figure 6:**
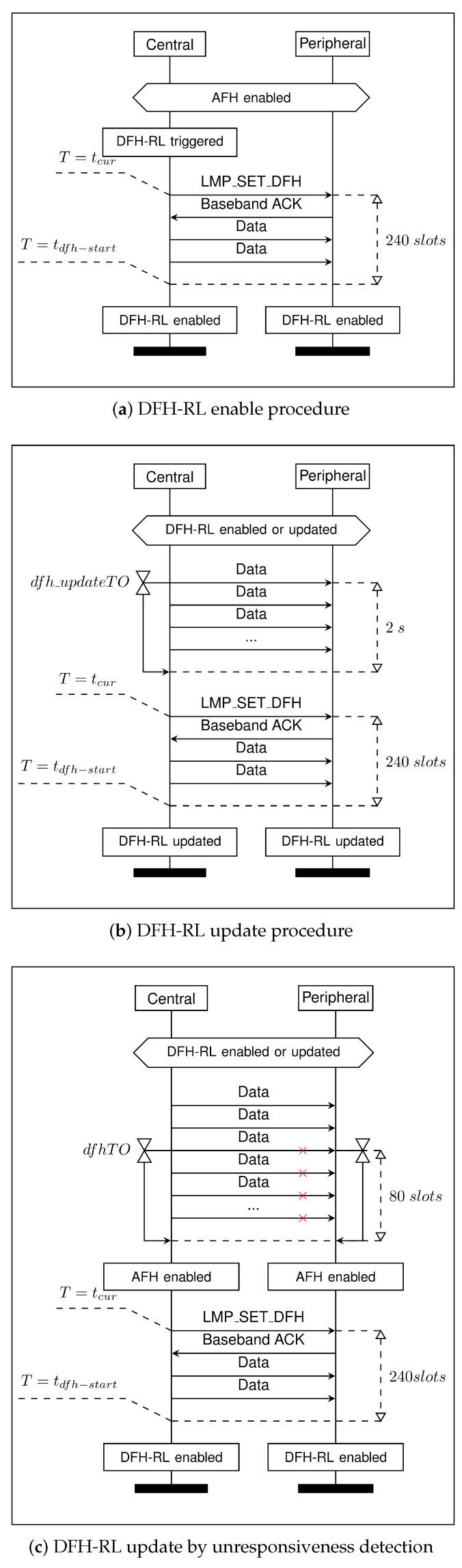
DFH-RL procedures.

Reflecting these implementation considerations, DFH-RL continuously executes the following procedures, as shown in Figure 6:

(a) Start from AFH and initiate DFH-RL by synchronizing the next best channel between the central and peripheral.
(b) Update the best channel information from the Q-table every 2 s.
(c) Switch to AFH and perform a best channel update if the peripheral fails to respond over two polling intervals (equivalently 80 slots, with a default polling interval of 40 slots). Once best channel synchronization is complete, enable diffusive hopping.

To support these procedures, we define a new control Protocol Data Unit (PDU) that is transmitted only by the central. We refer to it as LMP_SET_DFH, which contains two parameters: DFH start time ($t_{dfh-start}$) and DFH best channel ($CH_{bst}$).

- $t_{dfh-start}$ specifies when the best channel switch occurs, setting 240 slots (160 ms) in the future based on the central’s clock.
- $CH_{bst}$ is the best channel selected from the Q-table, used once DFH is enabled.

The central updates the Q-table based on transmission results and assigns the best channel to $CH_{bst}$ when generating LMP_SET_DFH. Upon receiving it, the peripheral compares $t_{dfh-start}$ with the current Bluetooth clock ($t_{cur}$). If $t_{dfh-start}$ has already passed, the best channel is immediately updated, and DFH is applied. Otherwise, a timer is set to apply the update when it expires. The central assumes the peripheral has completed the DFH parameter update once it receives a baseband acknowledgment and $t_{dfh-start}$ has passed. LMP_SET_DFH is generated every dfh_updateTO=2 s, based on the last update of $CH_{bst}$. If the central does not receive a baseband acknowledgment for data transmission or polling within two polling intervals (dfhTO=80 slots), it considers the peripheral unresponsive. In such cases, the system switches to AFH and immediately executes the DFH enable procedure (Figure 6c). This process updates the central’s Q-table, delivers a new best channel to the peripheral, and transitions the system back to DFH.

### 4.3. Prototype Implementation

**Figure 7:**
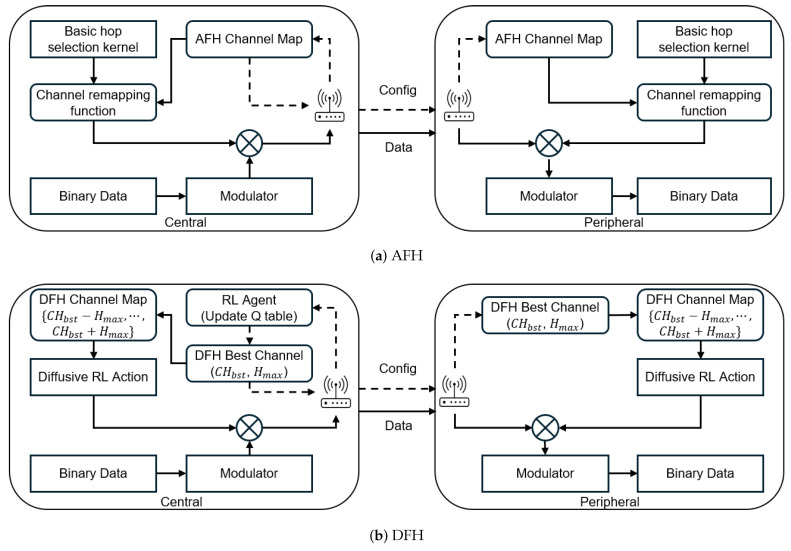
Operation of AFH vs. DFH in the prototype implementation.

To evaluate the proposed approach, we developed proof-of-concept (POC) prototype systems on a test device using the LG2630 [31], a Bluetooth dual-mode controller component developed and certified by LG Electronics. We modified the Link Manager to implement the LMP procedure for DFH-RL and adjusted the Link Controller software for frequency changes within the Bluetooth protocol stack, while keeping the hardware unchanged by leveraging the frequency setting mechanism defined in the Bluetooth standard Test Mode. AFH was modified to operate based on the PDR metric, and both schemes were implemented on the test device to compare collision probabilities. Figure 7 illustrates the operation of our implementation. In conventional AFH, as shown in Figure 7a, the Basic Hop Selection Kernel (BHSK) determines the pseudo-random frequency for each transmission. If the selected frequency falls on an unused channel, the channel remapping function redirects it to an available channel. To enable this process, AFH requires channel map synchronization. To maintain synchronization, the central device periodically generates control messages containing at least 20 used channels, ensuring consistency across the network. A channel is classified as bad if significant packet loss is detected. To simplify evaluation and ensure objectivity, we assume the channel measurement technique relies solely on the packet delivery ratio (PDR). Alternative methods, such as Received Signal Strength Indicator (RSSI), sniffing, or passive monitoring are excluded from this study because they can be influenced by hardware performance and transmission power.

In contrast to AFH, DFH-RL (Figure 7b) employs an RL Agent within the Link Manager, updating the Q-table upon receiving ARQ feedback (i.e., ACK/NACK) from the baseband. At each decision epoch—corresponding to the moments requiring the DFH Update, as illustrated in Figure 6b,c—the best channel $CH_{\mathrm{bst}}$ is selected as the one with the highest Q-value. The DFH channel map is locally generated by including channels within [$CH_{\mathrm{bst}}-H_{\max}$, $CH_{\mathrm{bst}}+H_{\max}$] using wrap-around indexing. The central device transmits only the best channel index to the peripheral using LMP_SET_DFH, enabling identical channel map generation without sending the entire hop set, thereby reducing synchronization overhead. The number of channels in the DFH map is thus 4 to 16 times fewer than in AFH, significantly reducing collision probability while maintaining minimal synchronization overhead. The Diffusive RL Action module applies an epsilon-greedy (ϵ-greedy) strategy during each transmission, enabling pseudo-random exploration and exploitation of the best channel neighborhood to optimize frequency hopping patterns. This adaptive approach significantly improves interference avoidance while maintaining minimal synchronization overhead.

## 5. Performance Evaluation

In this section, we evaluate DFH’s performance against AFH through simulations as well as real-world measurements using the prototype system of AFH and DFH.

### 5.1. Simulation

We implemented the procedures in Figure 6 as well as Algorithm 1 in a simulator implemented in C to evaluate DFH-RL (simulation codes are available at https://github.com/giwone/DFH, accessed on 18 September 2025.). All piconets share synchronized slot timing, such that channel changes occur at the same time across devices. However, data transmission starts at a random time within the first two seconds of the experiment for each piconet, and all timers introduced in Section 4.2 are initialized with respect to each piconet’s transmission start time. Measurements began immediately after this initial period. We did not consider channel conditions or hardware characteristics, and we assumed that all piconets are within the communication range defined by the disc model, where packet loss occurs whenever multiple transmissions overlap in time on the same channel, and all transmissions succeed if there is no overlap. We considered only the one-slot data transmission case. In this model, the central transmits an 83-byte payload using the 3-DH1 packet type (maximum payload size: 83 bytes) in every even-numbered slot, and the peripheral responds with an ACK or NACK in every odd-numbered slot. The simulation environment is summarized in Table 5. For comparison, we also implemented the following frequency hopping schemes, as shown in Algorithm 2:

- Legacy frequency hopping (LFH);
- Legacy frequency hopping with RL (LFH-RL);
- Adaptive frequency hopping (AFH);
- Adaptive frequency hopping with RL (AFH-RL).

LFH serves as the baseline, selecting the next channel uniformly at random from the available channel space. For uniform randomness, we use the Basic Hop Selection Kernel (BHSK) in Bluetooth v5.4, modified to allow variation in the number of channels *C*. BHSK used in LFH is a pseudo-random hopping sequence generator that computes the next hop channel index via polynomial and modulo operations based on the central device’s Bluetooth address and native clock. It computes the next channel to hop to, f(k), as follows: $f\left(k\right)=k+CLK_{27-2}\;\mathrm{mod}\;79,$ where $k=perm5(A_{LAP}⊕CLK_{16-12})$ is a pseudo-random number based on the lower 24 bits of the master device address $A_{LAP}$, perm5() is a 5-bit permutation function based on the LAP, and $CLK_{a-b}$ is the bits *a* through *b* of the Bluetooth clock. Regardless of channel conditions or packet collisions, it uses the channel corresponding to the computed index from among the *C* channels numbered 0 through C−1 for every transmission. LFH-RL is a hypothetical scheme that builds on LFH by incorporating RL to exploit the least-interfered channels. However, for exploration, LFH-RL retains the legacy uniform random hopping method over the entire channel space, similar to LFH. It is compared with DFH-RL to assess how much of DFH-RL’s performance gain stems from diffusive hopping rather than the RL component. Additionally, we apply the same supplementary mechanisms developed for DFH-RL to LFH-RL. For AFH, we implemented the standard procedure for channel map updates. Channel hopping follows the adaptive hop selection kernel, which modifies the BHSK with a channel remapping function. However, because channel measurement metrics for updates are vendor-specific, we use the packet delivery ratio (PDR) as the metric in this study. PDR is a reliable indicator for channel measurement and exclusion [32]. AFH maintains the best channel set *B* of size $\left|B\right|=C_{afh}<C$, where *C* is the total channel space size, based on the PDR metric. Similarly to LFH-RL, AFH-RL integrates reinforcement learning into its underlying hopping scheme. However, unlike LFH-RL, during exploration AFH-RL limits its random hops to the adaptively updated the best channel set *B*, instead of the full channel set. Algorithm 2 describes the five frequency hopping schemes in pseudocode. The simulation followed the RUN_RL procedure in Algorithm 1, varying *C* (number of channels) and $N_{a}$ (number of piconets) as defined in Table 5, and was executed for over 10,000 rounds for each scheme in Algorithm 2. For LFH and AFH, which do not employ RL, the CAL_REWARD and UPDATE_Q_TABLE procedures were omitted.

**Figure 8:**
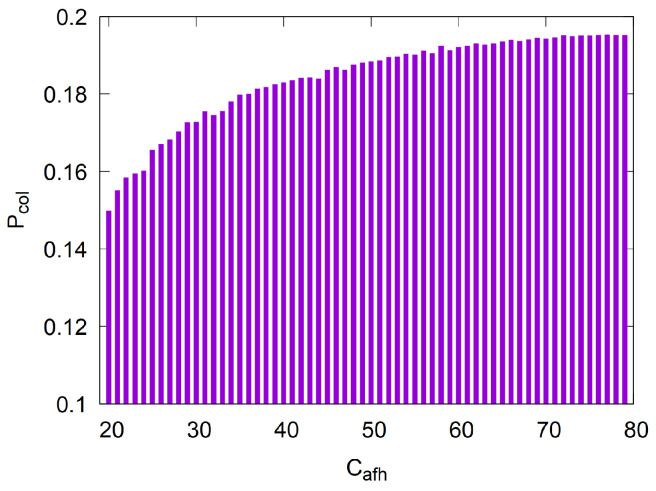
The collision probability in AFH for n=10 piconets, C=79 channels, and $C_{afh}$ = [20:79] used channels (rounds = 100,000, 95% CI ≤±0.00245).

In the following simulation experiments, we use the configuration in Table 5. In particular, we set $C_{afh}$ to its minimum possible value of 20, as this most effectively minimizes interference between piconets within the channel space. For example, Figure 8 presents the simulated collision probability of AFH when each piconet select only $C_{afh}<C$ channels, even when all *C* channels are available. The results clearly show that reducing $C_{afh}$ decreases the collision probability.
**Algorithm 2** Frequency hopping schemes.  1:**procedure** select_legacy_action(*a*)                   ▹*a*: agent  2:      return basic_hop_selection_kernel(C)            ▹*C*: no. of channels  3:**end procedure**  4:** **  5:**procedure** select_legacy_RL_action(*a*)  6:      **if** $rand\left(\right)/R_{max}<\epsilon_{lfh}$ **then**                    ▹ Explore  7:            return basic_hop_selection_kernel(C)  8:      **else**                              ▹ Exploit  9:            best←i **s.t..** $\forall_{j\leq C}\;a.Q\left[i\right]>a.Q\left[j\right]$                ▹*Q*: Q-table10:            return best11:      **end if**12:**end procedure**13:** **14:**procedure** select_adaptive_action(*a*)15:      selected_channel←basic_hop_selection_kernel(C)16:      **if** selected_channel∉B **then**17:            i←channel_remapping_function(B)18:            return B[i]19:      **else**20:            return selected_channel21:      **end if**22:**end procedure**23:** **24:**procedure** select_adaptive_RL_action(*a*)25:      **if** $rand\left(\right)/R_{max}<\epsilon_{dfh}$ **then**                    ▹ Explore26:            selected_cahnnel← select_adaptive_action(*a*)27:            return selected_channel28:      **else**                               ▹ Exploit29:            best←i **s.t..** $\forall_{j\leq C}\;a.Q\left[i\right]>a.Q\left[j\right]$30:            return best31:      **end if**32:**end procedure**33:** **34:**procedure** select_diffusive_RL_action(*a*)35:      best←i **s.t..** $\forall_{j\leq C}\;a.Q\left[i\right]>a.Q\left[j\right]$36:      **if** $rand\left(\right)/R_{max}<\epsilon_{dfh}$ **then**                    ▹ Explore37:            $dx\leftarrow rand\left(\right)\;\%\;H_{max}+1$               ▹dx: displacement38:            return (best+dx) or (best−dx)39:      **else**                               ▹ Exploit40:            return best41:      **end if**42:**end procedure**

Table 6 lists other assumptions that we have for our simulation experiments. Although we focus on these base assumptions, more generalized results for non-line-of-sight (NLoS) channels and denser deployment scenarios are discussed in Appendix A. All simulation results are reported with 95% confidence intervals, computed over 10 independent runs.

**Figure 9:**
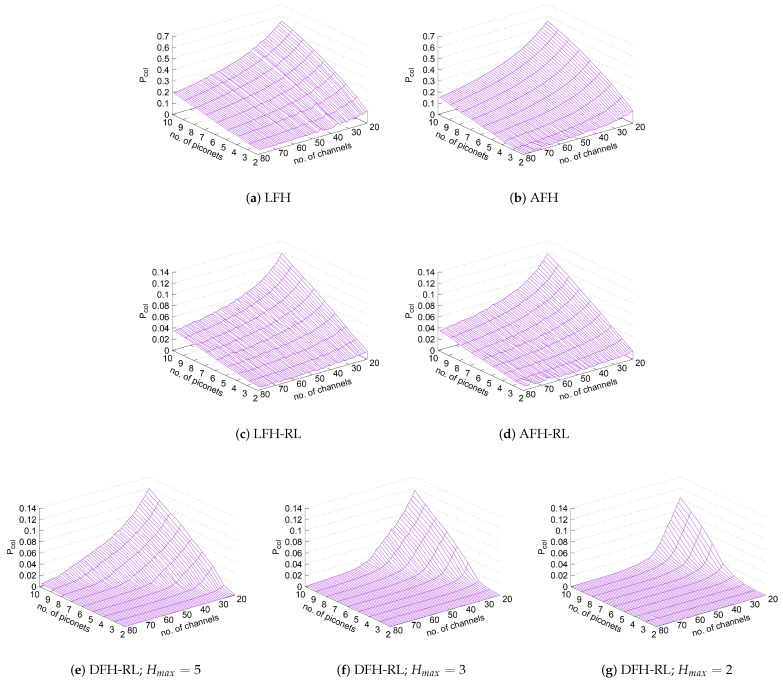
Collision probability comparison for various hopping schemes with respect to the number of piconets and channels (round = 100,000, 95% CI ≤±0.0031).

Figure 9 compares the packet collision performance of the evaluated schemes over 100,000 rounds, including DFH-RL at three diffusivity levels. LFH results in an excessively high packet collision probability per transmission under high channel contention. For instance, with n=10 piconets and only C=20 channels, the collision probability approaches 60%. Even with all 79 channels, $P_{col}\approx 20$% for n=10 piconets, and the performance would further deteriorate with fewer available channels. AFH offers only a marginal improvement over LFH, particularly in low-contention scenarios with fewer piconets and a larger channel space. However, it fails to provide effective protection against self-interference.

The introduction of RL seems to significantly improve performance for all compared schemes. In particular, LFH-RL achieves a substantial reduction in $P_{col}$ compared to LFH and even AFH. In the worst case, with C=20 and n=10, $P_{col}\approx 0.11$, a fivefold reduction compared to LFH. However, there is caveat to the contribution of RL for LFH and AFH. When every node keeps randomly jumping from channel to channel with maximum freedom, there cannot be any “learned memory” from RL because there is no particular channel to memorize to be better than other channels. They are all the same, with the same probability of collision. What RL looks to achieve for LFH or AFH, therefore, is in fact what reduced diffusivity achieves, thanks to a small probability of exploration (ϵ≪1) as opposed to large probability of exploitation (1−ϵ). In essence, their good performance under RL is a pure product of a small exploration probability, not that of the learned memory that is typically expected in RL-based algorithms.

Based on this fundamental observation, what DHF-RL achieves in addition to simply employing RL is even further limit the channel hopping to a neighborhood of the currently occupied channel, i.e., diffuse, so that the “memory” of bad channels where other nodes probably dwell finally becomes meaningful as in a typical RL-based algorithm. This is why DFH-RL collision probability can approach zero under most piconet population and channel space sizes. In contrast, AFH and LFH cannot achieve this result due to their fundamentally large diffusivity in hopping and no learned memory of channel use pattern.

In DFH-RL, diffusivity is further reduced spatially by decreasing Δx. During exploration, the hopping range is constrained to $H_{max}$ (lines 36–38) rather than spanning the entire channel space, providing an additional performance boost. The benefits of reduced spatio-temporal diffusivity emerge first in less contentious environments, where fewer piconets and larger channel spaces reduce interference. However, as diffusivity decreases further, the performance gain extends to more contentious environments as well. Notably, with $H_{max}=2$, $P_{col}$ remains close to zero in most cases, except in scenarios with small channel spaces and large piconet populations. These results confirm that diffusion is highly effective in mitigating packet collisions.

**Figure 10:**
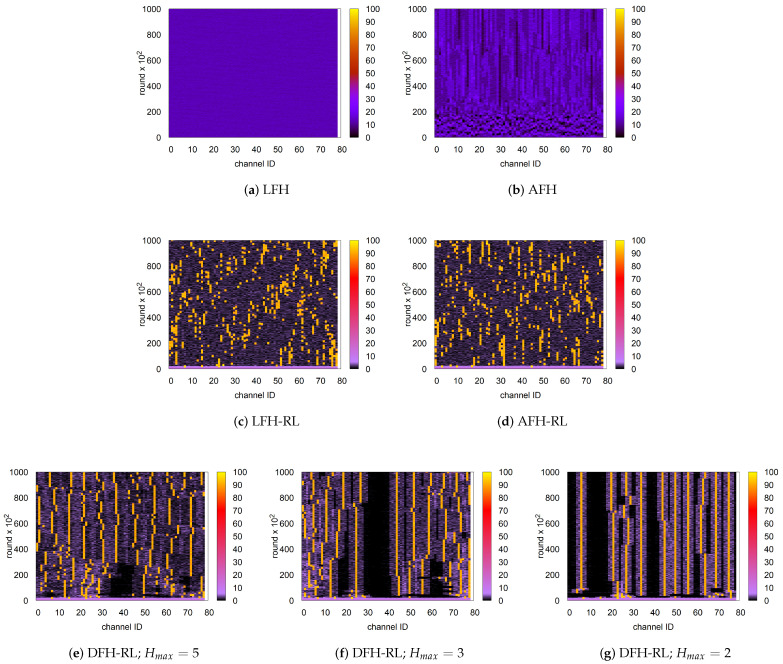
Channel visit frequency over time for n=10 piconets and C=79 available channels (rounds = 100,000; y-axis: time from bottom to top). The color of each cell, representing a 100-round time slice, indicates the number of visits to the corresponding channel (0–100). (**a**–**d**) LFH and AFH without and with RL. (**e**–**g**) DFH-RL ($H_{\max}=5,3,2$): Persistent, selective usage; smaller $H_{\max}$ yields tighter clustering and less interference without explicit hop set partitioning.

Finally, an RL-enhanced AFH hardly shows improvement over LFH-RL. Since both schemes retain the same uniform random hopping characteristic and differ only in the size of the exploration set, their effective diffusivity remains unchanged. This is confirmed by the similar channel visit frequency over time in Figure 10c,d.

Table 7 shows the collision probability values for the four corners of each graph in Figure 9.

Figure 10 visualizes how DFH-RL achieves its performance gain over the other three schemes. It illustrates channel utilization over time (bottom to top) for n=10 and C=79. LFH exhibits highly uniform channel visitation across the entire space. In contrast, AFH develops preferential visit patterns that stabilize after an initial perturbation, as piconets establish their niches. However, due to the large number of piconets (n=10), these subspaces significantly overlap, leading to the relatively marginal performance improvement observed in Figure 9. Compared to LFH and AFH, LFH-RL generates a more exploitative pattern guided by RL, concentrating its channel footprint on certain frequencies. However, no particular channels remain consistently preferred over time. Finally, DFH-RL demonstrates highly selective and persistent channel usage patterns after a brief initial perturbation, particularly with smaller $H_{max}$ values. By doing so, the diffusive scheme effectively minimizes interference among piconets compared to unrestricted uniform random hopping. Notably, DFH-RL achieves this without explicit hop set assignments. Notably, as observed in Figure 10e–g, larger $H_{\max}$ values spread footprints into available empty spaces more effectively. An optimal $H_{\max}$ may exist depending on network density and channel availability. This possibility requires further investigation.

### 5.2. Scalability to High-Density Environments

In Section 5.1, we demonstrated DFH-RL’s core performance across a range of scenarios with up to 10 piconets. To further validate our approach and a key theoretical prediction, its scalability, we now extend our evaluation to high-density network environments with up to 50 piconets. As the number of competing piconets grows, conventional methods like AFH struggle to mitigate self-interference. This section provides a detailed analysis of how DFH-RL’s collision mitigation capabilities scale to accommodate a larger number of piconets, offering critical evidence of its robustness in increasingly crowded wireless spaces. The simulation environment and parameters are identical to those in Table 6.

**Figure 11:**
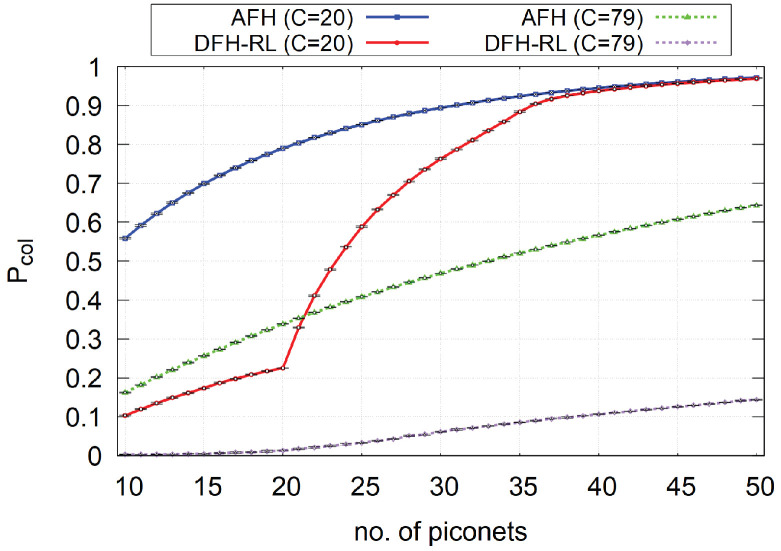
Comparison of collision probability between AFH and DFH-RL for *n* = [10:50] piconets, *C* = [20:79] channels, and $H_{max}=2$. The plot shows the 95% confidence interval from 10 repetitions of each experiment with 100,000 rounds.

As shown in Figure 11, collision probability ($P_{col}$) increases as the number of piconets grows from 10 to 50. This trend is expected due to higher network density and is consistent with results for fewer than 10 piconets. A notable phenomenon appears for DFH-RL in the channel-constrained case (C=20). While $P_{col}$ still remains lower than AFH, it rises sharply once the number of piconets exceeds this threshold. This behavior reflects the core mechanism of DFH-RL, which concentrates on exploiting a limited set of “best” channels. When the number of piconets approaches the number of available channels (C=20), unique optimal channel allocation becomes impossible, leading to contention and a rapid increase in collisions. By contrast, when more channels are available (C=79), DFH-RL sustains a near-zero collision probability, demonstrating its high efficiency in unconstrained environments.

### 5.3. Proof-of-Concept Prototype DFH Device

**Figure 12:**
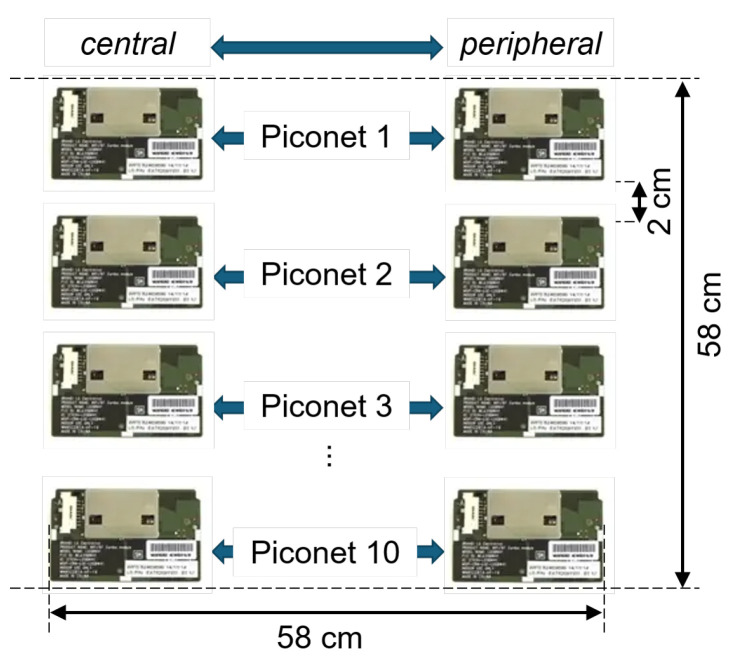
Proof-of-concept prototype test setup.

We evaluated the performance of the prototype DFH-RL system in a shielded room. As shown in Figure 12, we set up 10 piconets, each consisting of one test device as the central and another as the peripheral. To maximize channel contention, the piconets were placed literally next to each other. The parameter configuration matched that used in Table 5.

**Figure 13:**
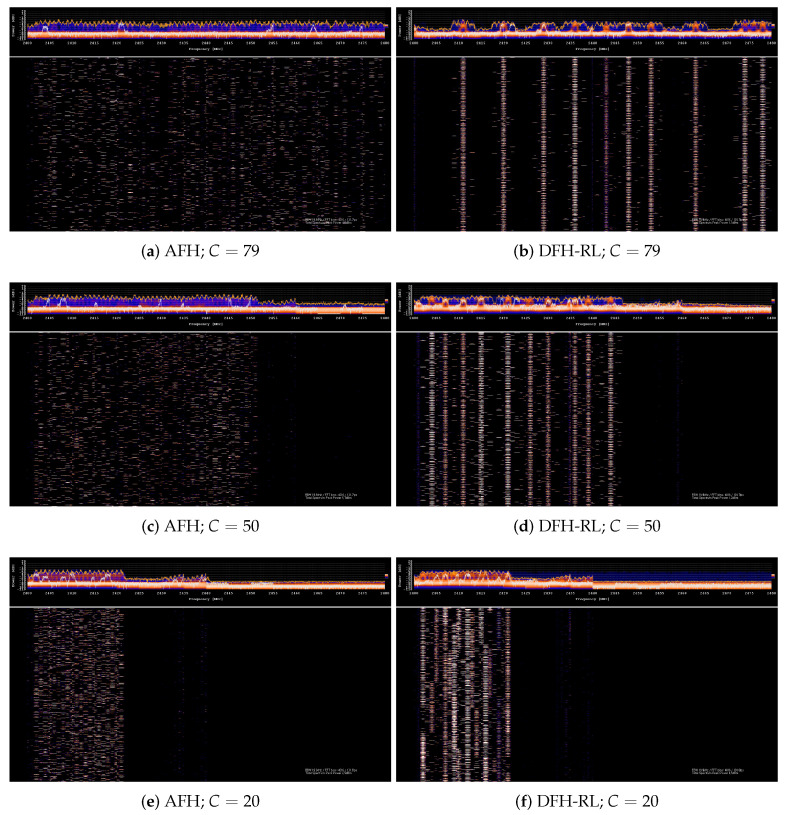
Spectrum analyzer output showing the channel-hopping behavior of AFH and DFH-RL in the POC prototype with n=10 piconets. Experiments were conducted with C=79,50, and 20 available channels; for DFH-RL, $H_{\max}=2$. The vertical axis represents time (top = newest, bottom = oldest), the horizontal axis represents frequency, and brightness indicates signal strength. (**a**,**c**,**e**): AFH shows widespread channel usage with a uniform random hopping pattern. (**b**,**d**): DFH-RL exploits the best channel and explores within $H_{\max}=2$, resulting in 10 distinct patterns corresponding to each piconet. (**f**): Changes appear after a best-channel update.

Figure 13 illustrates the behavior of DFH-RL and AFH with 10 competing piconets at C=20,50,79, observed through a spectrum analyzer based on HackRF r4 software-defined radio (SDR) [33]. The vertical axis represents time, while the horizontal axis represents frequency. AFH exhibits a uniformly random scattering of footprints, whereas DFH-RL piconets remain within their neighborhood unless a collision happens. As contention increases with a smaller channel space (e.g., at C=20), movements caused by collisions and subsequent Q-table updates become more dynamic. These real-world measurements confirm that the implemented AFH and DFH-RL operate as designed.

**Figure 14:**
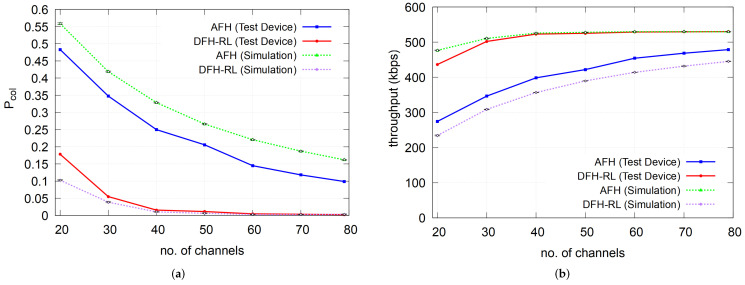
Comparison of collision probability and throughput between AFH and DFH-RL: prototype measurements vs. simulation experiments with 95% confidence interval; n=10, *C* = [20:79], $H_{max}$ = 2. (**a**) Collision probability. (**b**) Throughput: The maximum achievable throughput with the 3-DH1 packet type, in the absence of collisions, is 531.2 kbps.

Figure 14 compares the packet collision and throughput performance observed in the simulation and the prototype test. The prototype AFH exhibited a lower collision rate than the simulation, which could be attributed to several factors. In the simulation, any overlapping data transmission by multiple piconets is strictly considered a collision, whereas in real-world conditions, this is not always the case. Two unsynchronized time slots used by different piconets may overlap only in their unused portions, avoiding actual collisions. Conversely, the DFH-RL simulation tends to underestimate the collision rate due to its more static channel selection behavior. Unlike AFH, DFH-RL can experience consecutive collisions if the Q-table is not updated frequently enough to demote a collision-prone channel from its best-channel status. In our prototype, the update interval was set to two seconds, following many AFH implementations that update the channel map at similar intervals. During this period, DFH-RL may experience consecutive collisions, especially under high contention (e.g., at C=20). If collisions persist for two poll intervals (i.e., 80 ms), the system switches to AFH, as shown in Figure 6c. However, both consecutive collisions and the transient AFH operation, which has a higher collision rate than DFH-RL, lead to a higher average collision rate in the prototype than in the simulation, where updates occur more frequently. As channel contention decreases, this prediction gap diminishes. Overall, the gap between the simulation and the POC performance is due to the inaccuracy of the simulation model. A thorough study to reconcile simulation results and the POC performance is necessary. In particular, revising the simulation model will be a topic for further investigation.

Despite some discrepancies, the overall trend predicted by the simulation was reproduced in prototype tests. The results confirmed that DFH-RL consistently outperformed AFH across all scenarios. Even under the worst case of channel contention (C=20), DFH-RL achieved a collision probability $P_{col}\approx 0.18$, representing a reduction by a factor of 2.7× compared to AFH. Moreover, under this worst-case setting, the maximum achievable throughput with the 3-DH1 packet type was 531.2 kbps, while DFH-RL attained 436.54 kbps and AFH only 274.52 kbps, showing that DFH-RL achieved approximately 1.59× higher throughput than AFH. These results validate that the diffusive hopping behavior of DFH-RL in the prototype implementation aligns with the predictions from analysis and simulation in previous sections.

### 5.4. Performance Under Realistic Channel Conditions

Our previous simulations in Section 5.1 and Section 5.2 relied on idealized assumptions, such as a logical collision model that ignores attenuation and multipath fading. To validate whether these idealized assumptions are a reasonable proxy for real-world performance, we conducted a more comprehensive analysis. We extended our simulation to include both realistic channel conditions and denser passenger environments. We modeled realistic channel conditions by applying a Rayleigh fading model to reflect non-line-of-sight (NLoS) conditions common in dense deployments like subways. The simulation parameters for this are detailed in Table 8.

For environmental modeling, we simulated a crowded scenario with a subway congestion level of 150%, which corresponds to an average inter-person spacing of 46 cm (Table 9). This value was applied as the minimum separation between piconets, which were then randomly placed in the simulation space. Although the number of simulated piconets was fixed at 10, density was varied by shrinking the simulation area. We calculated the effective area for a larger hypothetical set of piconets (24 or 48 at 150% density) and placed the fixed 10 piconets within that space. Here, the term piconet-to-passenger ratio refers to the fraction of passengers assumed to operate independent Bluetooth piconets. The simulation width was kept constant at 3.1 m.

- 10% Piconet-to-Passenger Ratio: A total of 10 piconets in a 6.87m×3.1m area, equivalent to 24 piconets at 150% density.
- 20% Piconet-to-Passenger Ratio: A total of 10 piconets in a 3.43m×3.1m area, equivalent to 48 piconets at 150% density.

**Figure 15:**
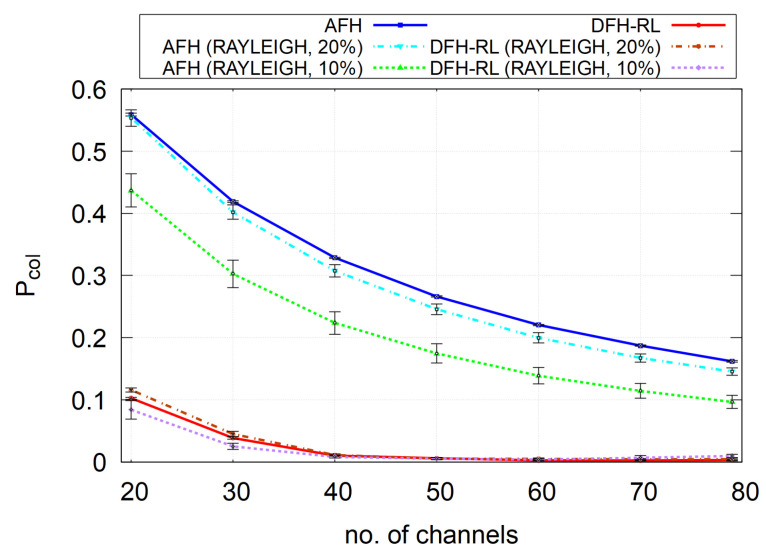
Comparison of collision probability with and without a channel fading model. Solid lines indicate results without fading, while dotted lines show results with a Rayleigh fading model. Labels (RAYLEIGH, 10%) and (RAYLEIGH, 20%) denote simulated environments with 10% and 20% piconet-to-passenger ratios, respectively. Each curve represents the average of 10 runs, each consisting of 100,000 rounds; parameters: *n* = [10:25] piconets, *C* = [20:79] channels, and $H_{max}=2$.

Incorporating these realistic factors into our simulation, Figure 15 shows that the performance of DFH-RL under Rayleigh fading in the “20% Piconet-to-Passenger Ratio” scenario closely matched the logical collision model. In contrast, the “10% Piconet-to-Passenger Ratio” scenario resulted in a smaller packet collision probability for AFH. The fact that the performance in the saturated “20% Piconet-to-Passenger Ratio” scenario aligns with our simplified model suggests that the logical model effectively captures a saturated interference state, justifying its use as a performance benchmark in dense network deployments.

### 5.5. Performance Under Asynchronous Operation and Traffic

Beyond channel conditions, a key challenge in real-world Bluetooth deployments is the asynchronous nature of piconet operation. While our previous simulations assumed synchronized timing, a more accurate model must account for the independent slot phases of each piconet. This subsection extends the evaluation in two parts: (i) an asynchronous BR/EDR piconet timing and collision model, contrasted with a synchronized slot model and the prototype; (ii) a download (tethering) traffic model used to compare AFH and DFH-RL under multi-slot bursts.

#### 5.5.1. Asynchronous Piconet Timing and Collision Modeling

The baseline simulator considered slot-level collisions only. To better reflect real deployments, each piconet runs with an independent slot phase ϕ∼U[0,625μs) and collisions are decided by on-air interval overlap, including the slave’s one-slot NULL/ACK response. For EDR packets, airtime is computed as $t_{\mathrm{air}}\left(S\right)=72+54+5+11+\frac{8(S+4)}{r}+2\;\left[\mu s\right]$ (Basic Rate AC/HDR, guard, DPSK sync/payload/trailer); the slave NULL is 126μ s. The key definitions are summarized in Table 10.

**Figure 16:**
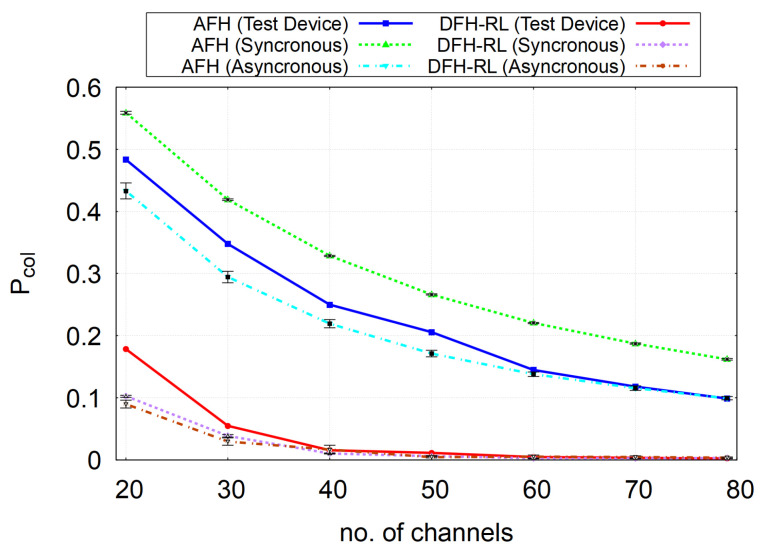
Comparison of collision probability (Async vs. Sync effect in simulation). Solid lines indicate prototype measurements; dotted lines indicate simulation results using the synchronous and asynchronous traffic models. Each curve represents the average of 10 runs, each lasting 100s; parameters: *n* = [10:25] piconets, *C* = [20:79] channels, and $H_{max}=2$.

The synchronized model aligns piconets to the same even/odd slot boundaries and uses the same airtime-based collision rule. Asynchronous phases reduce simultaneous starts; moreover, BR/EDR alternates a multi-slot master burst with a very short one-slot slave reply, leaving the remainder of that odd slot idle until the next even slot. As shown in Figure 16, this “burst + short reply + idle” cadence increases the chance that two piconets miss each other in time, shortening effective overlap on a hop and lowering $P_{\mathrm{col}}$ versus the synchronized case. Absolute values may still differ from the prototype since only time overlap is modeled (no fading/capture), but trends are closer to the prototype.

#### 5.5.2. Download Traffic Modeling (Tethering)

We model one-way file download with a downlink mixture as follows:

(3) DownlinkData:64B(15%)+TruncNormal(μ=1000,σ=120,[600,1021])(85%).

After each master TX block, the slave sends a one-slot response, which is a delayed-ACK (64B) in a 2:1 ratio, otherwise NULL (0B). The source is greedy (the next DL block starts at the next even slot). All experiments use EDR 3 Mbps (r=3).

**Figure 17:**
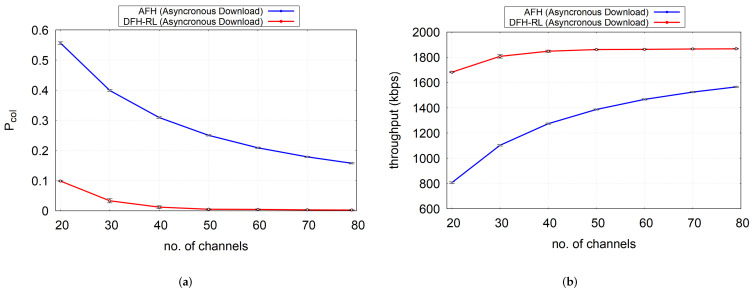
Comparison of collision probability and throughput between AFH and DFH-RL: asynchronous data transfer simulation experiments with 95% confidence interval; n=10, *C* = [20:79], $H_{max}=2$, DL: 64B (15%) +TruncNormal(μ=1000,σ=120,[600,1021]) (85%); UL: 1-slot response per block (ACK 64B with delayed-ACK 2:1, else NULL); slot mapping: ≤ 83B=1-slot, 84-552B=3-slot, 553-1021B=5-slot. (**a**) Collision probability. (**b**) Throughput: The maximum achievable throughput with the 3-DH5 packet type (3 Mbps, 5 slots), assuming no collisions, is 2178.1 kbps.

As shown in Figure 17a,b, with multi-slot (five-slot) download frames, AFH faces a higher chance of temporal overlap with other piconets, lengthening its collision exposure. Consequently, collisions rise and throughput falls relative to a one-slot burst pattern. By contrast, DFH-RL maintains a collision rate similar to the one-slot burst pattern and thus approaches the airtime-implied ceiling. For the downlink mixture above, the slot-based theoretical maximum throughput is ≈1.87Mbps. At C=30, DFH–RL achieves ≈1.8Mbps, whereas AFH attains only ≈1.1Mbps; i.e., DFH–RL provides about 1.64× (64%) higher throughput. Under realistic download traffic, multi-slot exposure penalizes AFH, whereas DFH-RL maintains lower collisions and near-max throughput.

### 5.6. CPU Energy Analysis

This section compares the CPU computational energy of the DFH-RL algorithm with that of the standard AFH algorithm. Since both algorithms operate under the same communication protocol, the primary difference in energy consumption arises from the CPU operations required for channel calculation. Therefore, this analysis focuses solely on the CPU computational energy ($E_{CPU}$).

The CPU computation energy is defined as follows:

(4) $$E_{CPU}=P_{CPU\text{\_}active}\times T_{CPU\text{\_}active}$$

The variables are defined as follows:

- $P_{CPU\text{\_}active}$ is the Average CPU Active Power. This is set to **7.26 mW**, a value derived from the official efficiency metric (101 CoreMark/mA) of the reference platform, the Nordic nRF5340 network core [34].
- $T_{CPU\text{\_}active}$ is the Total CPU Active Time, calculated as follows:
  (5) $$T_{CPU\text{\_}active}=\frac{C_{total}}{64\;\mathrm{MHz}}$$

In this context, $C_{total}$ represents the weighted sum of the dynamic instruction counts ($N_{i}$) and the Cycles Per Instruction (CPI) for each instruction class (see Table 11).

As mentioned in Section 4.2 of the main text, the instruction count for DFH is relatively higher because it invokes the Bluetooth hopping kernel for ϵ-greedy policy decisions and associated pseudo-random number generation. When exploration occurs with a probability of 0.1, a total of two hopping kernel calls are needed, leading to more instructions being executed on average compared to AFH. The dynamic instruction execution counts for a single channel calculation, obtained by analyzing the compiled binaries of each algorithm, are shown in Table 12.

Total execution cycles are computed as follows:

$$C_{\mathrm{total},\mathrm{AFH}}=462\;\mathrm{cycles},\;C_{\mathrm{total},\mathrm{DFH}}=554.1\;\mathrm{cycles}.$$

Assuming a communication scenario that requires 100 channel calculations per second, the resulting CPU computation energy is calculated in Table 13.

DFH-RL consumes 1.05 μJ/s more than AFH, corresponding to 1.05 μW of additional power. Compared to ≈15,000 μW (based on approx. 4 μA at 3.7 V [35]) during music playback, this is only 0.007%. Over 5 h of playback, total time is reduced by only ≈1.3 s, confirming negligible impact. DFH-RL requires ≈20% more CPU energy than AFH, but the absolute rise (1.05 μJ/s) is trivial relative to device power, making it a reasonable trade-off for improved stability. Limitations include the use of estimated CPI values and the omission of cache/pipeline effects, but the analysis sufficiently demonstrates the relative efficiency gap.

## 6. Properties of Diffusive Frequency Hopping

In this section, we further investigate the properties of DFH-RL through extensive simulation. Specifically, we examine its fairness toward AFH, its resilience to static interference (e.g., Wi-Fi), and its defense against narrow-band attacks.

### 6.1. Friendliness to AFH

**Figure 18:**
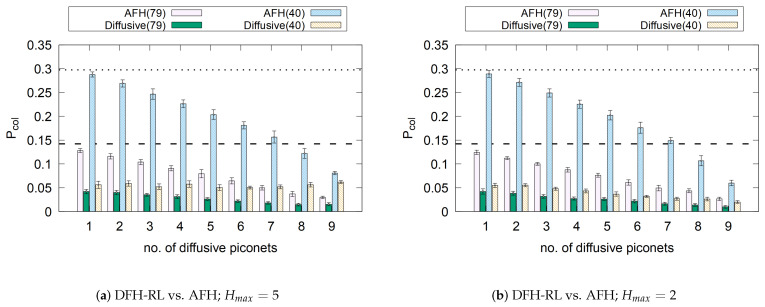
Collision probability with a 95% confidence interval in the mixed population of DFH-RL and AFH with n=10 piconets, C=79 and C=40, for (**a**) $H_{\max}=5$ and (**b**) $H_{\max}=2$. The number of DFH-RL piconets is varied from 1 to 9, with the remaining operating under AFH. Dashed lines indicate the collision probability when all 10 piconets operate under AFH with C=79, and dotted lines for C=40.

While diffusive frequency hopping clearly reduces packet loss when adopted by all piconets, a key question is whether AFH piconets are disadvantaged when operating near DFH-RL piconets. To investigate this, we set the number of diffusive piconets to *x* and the number of adaptive piconets to 10−x, where 1≤x≤9. Figure 18 presents the results, with dashed lines representing the packet collision rate for a pure AFH population using 79 channels and dotted lines for 40 channels. Interestingly, AFH piconets benefit from having DFH-RL neighbors. By using a smaller channel footprint, DFH-RL frees up more of the channel space for AFH piconets, reducing their collision rate. As the proportion of DFH-RL piconets increases, they not only mitigate self-interference but also provide cleaner channels for AFH piconets. These findings confirm that diffusive frequency hopping coexists harmoniously with existing Bluetooth piconets, enhancing overall network performance.

### 6.2. Coping with Static Interference from Wi-Fi

**Figure 19:**
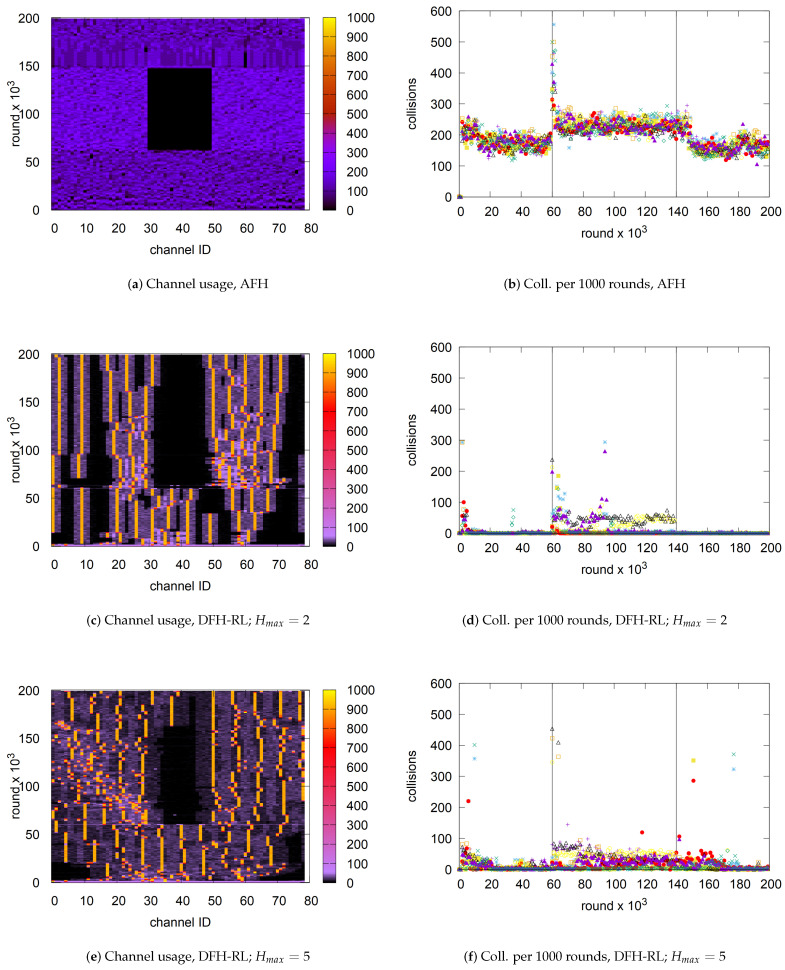
Channel visit frequency under Wi-Fi interference for n=10 piconets and C=79 available channels (rounds = 200,000). A 20 MHz Wi-Fi band (channels 31–50) is fully blocked from rounds 60,000 to 140,000. (**a**,**c**,**e**) Show channel visit frequency; (**b**,**d**,**f**) show collisions per 1,000 rounds, where each marker shape denotes one of the 10 piconets, for AFH, DFH-RL ($H_{\max}=2$), and DFH-RL ($H_{\max}=5$), respectively.

AFH was originally designed to mitigate static interference from Wi-Fi. Here, we evaluate whether DFH-RL can similarly handle Wi-Fi interference while also managing self-interference. The simulation environment used in Section 5.2 remained unchanged, and the parameters in Table 5 were set to C=79 and $N_{a}=10$. The simulation was run for a total of 200,000 rounds. In this experiment, we introduce a total packet loss condition over a 20 MHz band in the middle of the channel space (i.e., channels 31 to 50) and observe its impact on DFH-RL’s behavior and performance. As shown in Figure 19, static interference begins at 60,000 rounds and ends at 140,000 rounds. To accurately model AFH behavior under static interference, we used a signal generator to interfere with two recent smartphones (Z Flip 3 and iPhone 14) in a shielded room and monitored their channel usage. Through energy detection, both phones vacated the occupied Wi-Fi channel—along with small adjacent guard bands—within one second. Upon Wi-Fi deactivation, they returned to the released channel conservatively, taking 15 to 30 s. We replicated this AFH behavior in our simulator, except that the return to the released channel was immediate. In this setting, AFH was configured to exclude all channels that overlapped with the Wi-Fi channel from its channel map and to restore them immediately once Wi-Fi was turned off. In contrast, DFH-RL, without any dedicated monitoring, regarded transmissions on channels overlapping with the Wi-Fi channel as normal packet collisions. Meanwhile, self-interference was monitored through packet collisions in both AFH and DFH-RL.

From the channel usage patterns in Figure 19a,c,e, we observe that both AFH and DFH-RL vacate Wi-Fi-occupied channels immediately upon interference. In AFH, this evacuation is triggered by channel monitoring, while in DFH-RL, it is guided by Q-table values. In particular, three DFH-RL piconets operating within the affected channels migrate in Figure 19c,e. Figure 19b,d,f presents the corresponding collision counts per 10,000 rounds for the 10 piconets (each marked distinctly). DFH-RL initially experiences a higher collision rate, particularly at the start and when static interference begins, until the Q-table stabilizes. However, collisions quickly subside once the learning process adapts. In contrast, AFH piconets consistently experience significantly higher self-interference, which worsens under static interference due to the reduced available channel space.

When static interference ends at the 140,000th round, AFH quickly reclaims the previously blocked channels. In contrast, DFH-RL tends to remain longer within the migrated channels. During exploitation, piconets return only if they experience collisions, causing the Q-values of their current channels to drop below those of the previously interfered channels. During exploration, they gradually reintroduce themselves into the vacated channels, as illustrated in Figure 19e. A potential way to accelerate this return is to integrate AFH with DFH-RL, allowing periodic channel map updates to influence the Q-table. We leave this for future work.

Although DFH-RL is slower in returning to the released channels, the number of collisions after the 140,000th round remains significantly lower than in AFH. Within a few seconds, the collision rate stabilizes to pre-interference levels. This is because piconets are evenly distributed, and their hopping ranges remain sufficiently isolated within the channel space. In summary, DFH-RL effectively mitigates static interference while incurring far fewer collisions than AFH.

### 6.3. Coping with Potential Narrow-Band Attacks

Since diffusive hopping operates within a small neighborhood of channels, one practical challenge is narrow-band attacks. While frequency hopping over a large channel space is no longer an effective countermeasure against eavesdropping [36], narrow-band jamming remains a concern. If the jamming is static and confined to a fixed band, our previous experiment demonstrated that diffusive hopping handles it as effectively as AFH. However, if the narrow-band attack is more dynamic, an additional countermeasure will be required.

**Figure 20:**
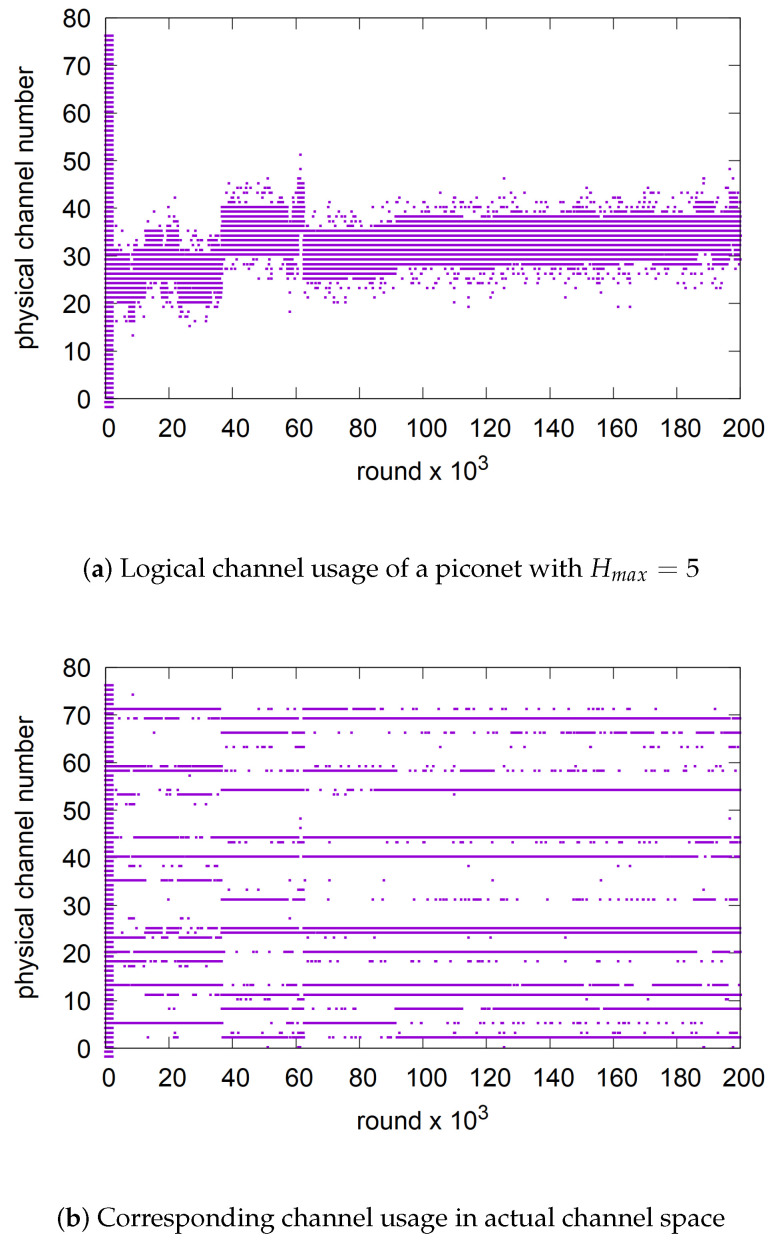
DFH-RL with and without maximum entropy permutation (example). Each purple dot represents the channel number selected in a given round, over a total of 200,000 rounds.

One approach to countering dynamic narrow-band attacks on diffusive hopping is to treat the ring topology in Figure 4 as logical and map it to a physical ring. For example, the Fisher–Yates shuffle [37] can be used to achieve a maximum entropy permutation in this logical-to-physical mapping. As long as both the central and peripheral map the logical ring to the same physical ring, DFH-RL retains its key properties while significantly mitigating the impact of narrow-band jamming. Figure 20 illustrates how the Fisher–Yates shuffle disperses the concentrated logical channel usage of diffusive frequency hopping across a broader range of physical frequencies. In Figure 20a, the logical diffusion with $H_{max}=5$ is spread across a much wider set of physical channels than in Figure 20b. This redistribution makes narrow-band jamming and eavesdropping far less effective compared to the non-shuffled DFH-RL.

## 7. Conclusions

This paper has introduced a diffusion-based perspective on random access and demonstrated, through analysis, simulation, and proof-of-concept prototyping with Bluetooth Classic, that diffusive frequency hopping combined with reinforcement learning (DFH-RL) can substantially mitigate self-interference relative to adaptive frequency hopping (AFH). In several constrained experimental settings, DFH-RL reduced collision probability by up to 63% and increased throughput by up to 1.5× compared to AFH. These results establish initial evidence that non-uniform random access may offer tangible benefits for dense environments. Future work will extend the evaluation of DFH-RL to other realistic traffic types, more dense deployments, and heterogeneous coexistence scenarios.

## Figures and Tables

**Table 1:** List of recent studies related to Bluetooth self-interference and related MAC approaches.

Study	Approach	Main Contribution	Limitations/Gaps
Poirot (2021) [19]	eAFH	Faster reintegration of excluded channels	Still pseudo-uniform; limited self-interference relief
Eltholth (2023) [18]	Chaotic hopping	Better coexistence with Wi-Fi	Gains mainly under extreme cases (40 piconets, 10 channels)
Wang (2023) [13], Li (2020) [14], Zhao (2024) [15]	Pattern-aware sensing	Detect patterned interference (radar, jamming) via exclusion or ML	Not tailored for Bluetooth; assumes structured interference
Atheeq (2024) [20]	Chaotic hopping	High unpredictability, resilience against jamming	Focused on security, not friendly piconet collisions
Ganipsetty (2024) [17]	Periodic non-uniform hopping	Improved SNR and BER	Rigid patterns; lacks adaptability
Hu (2025) [22]	DNMC (mesh)	Edge-assisted decentralized routing	Requires mesh infra; not Bluetooth-friendly
Rahman (2025) [23]	RL-based MAC	DQN adaptation for real-time channel access	Too resource-intensive for Bluetooth controllers
Xu (2025) [24]	Multi-objective MAC	Ensures fairness/min capacity in IoT	Needs central coordination/global knowledge

**Table 2:** Comparison of related studies by distinctive characteristics.

Approach	Core Principle	Collision Analysis	Optimization	Scalability
Conventional Heuristic Methods	Empirical Rules	Post Hoc, Empirical	Ad Hoc, Unsystematic	Lacks Analytical Support
Reinforcement Learning-based Methods	Learning Through Trial and Error	Reactive	Requires Training	Training Complexity Increases
Diffusion Theory + RL	Mathematical Modeling Based on Diffusion Theory	Predictive, Allows for Theoretical Analysis	Requires Training	Provides Analytical Scalability Insights

**Table 3:** Bluetooth central devices and features used in shielded room measurements.

Central Device	Codec	Length	Size (Bytes)	Utilization/Slot Format	Market Introduction
iPhone 6 (“i6”)	AAC, 256 Kb/s		605–668		September 2014
			(avg. 78 × 7–8 segments)	0.6/1	
iPhone 6 (“i6”)	VBR	23.57 ms	526–672	1.2/5–3.6/5	September 2014
Xperia Z3 (×2, “Z3”)	SBC	14.77 ms	672	4.6/5	September 2014
Galaxy S6 (×2, “S6”)	SBC	14.77 ms	612	4.2/5	April 2015
Xperia Z5 (×2, “Z5”)	SBC	14.77 ms	672	4.6/5	September 2015
Galaxy S7 (“S7”)	SBC	14.77 ms	612	4.2/5	March 2016
Galaxy S8 (“S8”)	AAC, 320 kb/s	23.22 ms	672	4.6/5	April 2017
LG Q9 One (“Q9”)	AAC, 320 kb/s	29.77 ms	676	4.6/5	February 2019
Galaxy S20 (“S20”)	VBR	29.77 ms	676	4.6/5	March 2020
LG Velvet (“V”)	AAC, 165 kb/s	23.22ms	505	3.5/5	May 2020
Z Flip 3 (“F3”)	AAC, 320 kb/s	23.22 ms	84–367	0.8/3–2.6/3	August 2021
	VBR		(avg. 335)	(avg. 2.4/3)	
			368–670	2.6/5–4.6/5	
			(avg. 424)	(avg. 3/5)	
	N/ACK	125 μs			
10 Piconets	Exclude iPhone6 (×2), S7, and Q9			(avg. 3.770/5)	
12 Piconets	Exclude iPhone6 (×2)			(avg. 3.815/5)	
14 Piconets				(avg. 3.815/5)	

**Table 4:** Relation between diffusion and Bluetooth frequency hopping.

Diffusion		Bluetooth Frequency Hopping
Particles	⟷	Piconets
Diffusion interval (*L*)	⟷	Channel space (e.g., 79)
Diffusion length (Δx)	⟷	Channel hopping distance
Unit time (Δt)	⟷	2 slot times (e.g., 1.25 ms)
Encounter	⟷	Packet collision

**Table 5:** Simulation parameters.

Parameter	Meaning	Value
*C*	No. of channels	[20:79]
$C_{afh}$	No. of used channels in AFH and AHF-RL	20
$\epsilon_{lfh}$, $\epsilon_{dfh}$	Exploration thresholds	0.1
	in LFH-RL, AFH-RL, and DFH-RL	
$H_{max}$	Max. diffusive hopping distance	[2:5]
$N_{a}$	No. of piconets	[2:10]
α	Learning rate in LFH-RL, AFH-RL, and DFH-RL	0.1
γ	Reward discount factor	0.9

**Table 6:** Other simulation assumptions.

Item	Description
Simulation model	Disc channel model; Packet loss when transmissions overlap in time on same channel
	Link loss effects were not considered
Traffic pattern	One-way burst data transmission from central to peripheral, 3-DH1 (83 bytes), 1-slot (625 μs)
Simulation duration	rounds = 100,000
Synchronization	Slot timing across piconets is fully synchronized

**Table 7:** Sampled collision probability values from Figure 9 at four corner points (*n*,*C*) for each hopping scheme, where *n* is the number of piconets and *C* is the total channel space size.

Hopping Schemes	n=2,C=20	n=2,C=79	n=10,C=20	n=10,C=79
LFH	0.109383	0.039003	0.556387	0.185800
LFH-RL	0.014437	0.005613	0.117861	0.033788
AFH	0.109383	0.001856	0.556387	0.148511
AFH-RL	0.013988	0.007449	0.116942	0.034737
DFH-RL; $H_{max}=5$	0.001399	0.000000	0.119460	0.004144
DFH-RL; $H_{max}=3$	0.000057	0.000001	0.116265	0.000657
DFH-RL; $H_{max}=2$	0.000025	0.000000	0.103321	0.000114

**Table 8:** Simulation parameters for Rayleigh fading in a crowded subway scenario.

Parameter	Value	Description/Rationale
Simulation area	16.5 m × 3.1 m	Effective passenger area, simplified from the official 19.6 m × 3.12 m car dimension.
Minimum separation distance	0.46 m	Reflects personal space in a “Crowded” (150%) scenario from Table 9.
Transmit power	4 dBm	Standard output power for Bluetooth Class 2 devices.
Path loss model	Log-distance	Standard model for wireless attenuation. Parameters: exponent (*n*) = 4.5, reference loss ($PL(d_{0})$) = 40 dB @ 1 m.
Fading model	Rayleigh	Models signal strength variations in a non-line-of-sight (NLoS) multipath environment.
Body attenuation model	‘Phone-in-pocket’ scenario	Applied to the main agent to simulate signal passing through the body. Parameters: distance = 1.0 m, additional loss = 15 dB.
Receiver: thermal noise power	−95 dBm	Standard thermal noise for a 1 MHz channel bandwidth.
Receiver: SINR threshold	7 dB	Minimum SINR required for successful packet demodulation for a typical Bluetooth data rate.

**Table 9:** Average distance between people by congestion level (based on a standard South Korean subway car with a passenger area of 51.5 m^2^).

Congestion level	No. of People	Area per Person	Avg. Distance	Description
30%	48	1.07 m^2^	1.03 m	Very sparse: ample personal space.
50%	80	0.64 m^2^	80 cm	Uncrowded: many seats still available.
100%	160	0.32 m^2^	57 cm	Nominal: all seats are occupied, standing room is comfortable.
150%	240	0.21 m^2^	46 cm	Crowded: physical contact begins as the space is similar to shoulder width.
200%	320	0.16 m^2^	40 cm	Very crowded: movement is difficult.
250%	400	0.13 m^2^	36 cm	Saturated: bodies are compressed, requiring people to turn sideways.

**Table 10:** Timing and airtime definitions for the asynchronous model (EDR at 3 Mbps, fixed r=3).

Item	Expression	Notes
Slot phase (asynchronous)	ϕ∼Uniform[0,625μs)	Independent per piconet; decorrelates starts and shortens overlaps
Slot mapping (packet type)	S≤83⇒1-slot (3-DH1); 84–552⇒3-slots (3-DH3); 553–1021⇒5-slots (3-DH5)	User payload → slot occupancy
EDR airtime	$t_{\mathrm{air}}\left(S\right)=72+54+5+11+\frac{8(S+4)}{r}+2\;\left[\mu s\right]$	Aceess code 72 + HDR 54 (GFSK); guard 5 + sync 11 + trailer 2 (DPSK)
Slave NULL airtime (BR)	126μs	72+54 only (no EDR fields)
Variables	*S*: user payload size (bytes); r=3 bit/μs	EDR at 3 Mbps fixed in all experiments

**Table 11:** Estimated CPI per instruction class.

Instruction Class	CPI	Rationale
ALU	1	Most integer operations are single-cycle on Cortex-M33
Multiply/Divide	1.5	Mix of mul/div cycles
Memory Access	2	SRAM access + pipeline delay
Branch	2	Pipeline refill overhead
Other	1	Simple system instructions

**Table 12:** Comparison of dynamic instruction counts for a single channel calculation.

Instruction Class	AFH	DFH-RL
ALU	145	155.6
Multiply/Divide	4	3
Memory Access	101	130.3
Branch	11	16.4
Other	87	100.6

**Table 13:** Estimated CPU energy (100 calculations/sec).

Metric	AFH	DFH-RL	Δ
Cycles/s	46,200	55,410	+9210
CPU Active Time	0.722 ms	0.866 ms	+0.144 ms
Energy (μJ/s)	5.24	6.29	+1.05 (+20%)

## Data Availability

The original contributions presented in this study are included in the article; further inquiries can be directed to the corresponding author.

## Appendix A. Fairness of DFH-RL

The proposed DFH-RL method’s fairness is evaluated by its performance consistency among piconets and its stability across multiple runs. The results in Table A1 demonstrate that the proposed DFH-RL scheme achieves a very high degree of fairness across different channel counts. Jain’s index remains close to 1.0 in all cases, while the Gini coefficient, standard deviation, and coefficient of variation (CV) stay close to zero. These complementary fairness metrics consistently indicate that resources are evenly distributed among piconets, with minimal imbalance or bias. Even under the most constrained scenario (C=20), the fairness values remain strong, confirming that DFH-RL provides equitable channel access regardless of network density.

**Table A1:** Fairness of DFH-RL; $H_{max}=2$ and n=10 piconets with *C* used channels. Uparrows mean higher values are better; downarrows mean lower values are better.

Fairness Metric	C = 20	C = 30	C = 40	C = 50	C = 60	C = 70	C = 79
Jain ↑	0.9997	0.9999	1.0000	1.0000	1.0000	1.0000	1.0000
Gini ↓	0.0101	0.0040	0.0019	0.0012	0.0007	0.0012	0.0004
Stddev. ↓	0.0161	0.0077	0.0034	0.0022	0.0013	0.0022	0.0007
CV ↓	0.0180	0.0080	0.0034	0.0022	0.0013	0.0022	0.0007
